# Isolation of quinic acid from dropped *Citrus reticulata* Blanco fruits: its derivatization, antibacterial potential, docking studies, and ADMET profiling

**DOI:** 10.3389/fchem.2024.1372560

**Published:** 2024-04-18

**Authors:** Heena, Sonia Kaushal, Vishaldeep Kaur, Harsh Panwar, Purshotam Sharma, Raman Jangra

**Affiliations:** ^1^ Department of Chemistry, Punjab Agricultural University, Ludhiana, Punjab, India; ^2^ Department of Dairy Microbiology, Guru Angad Dev Veterinary University, Ludhiana, Punjab, India; ^3^ Department of Chemistry and Centre for Advanced Studies in Chemistry, Panjab University, Chandigarh, India

**Keywords:** biofilm, *citrus reticulata*, docking, dropped citrus fruits, quinic acid, lactone

## Abstract

*Citrus reticulata* dropped fruits are generally discarded as waste, causing environmental pollution and losses to farmers. In the present study, column chromatography has been used to isolate quinic acid (1,3,4,5-tetrahydroxycyclohexane-1-carboxylic acid) from the ethyl acetate fraction of a methanol extract of citrus fruits dropped in April. Quinic acid is a ubiquitous plant metabolite found in various plants and microorganisms. It is an important precursor in the biosynthesis of aromatic natural compounds. It was further derivatized into 3,4-o-isopropylidenequinic acid 1,5-lactone (QA_1_), 1,3,4,5-tetraacetoxycyclohexylaceticanhydride (QA_2_), and cyclohexane-1,2,3,5-tetraone (QA_3_). These compounds were further tested for their antibacterial potential against the foodborne pathogens *Staphylococcus aureus*, *Bacillus spp*., *Yersinia enterocolitica*, and *Escherichia coli.* QA_1_ exhibited maximum antibacterial potential (minimum inhibitory concentration; 80–120 μg/mL). QA_1_ revealed synergistic behavior with streptomycin against all the tested bacterial strains having a fractional inhibitory concentration index ranging from 0.29 to 0.37. It also caused a significant increase in cell constituent release in all the tested bacteria compared to the control, along with prominent biofilm reduction. The results obtained were further checked with computational studies that revealed the best docking score of QA_1_ (−6.30 kcal/mol, −5.8 kcal/mol, and −4.70 kcal/mol) against β-lactamase, DNA gyrase, and transpeptidase, respectively. The absorption, distribution, metabolism, excretion, and toxicity (ADMET) analysis revealed that the drug-like properties of QA_1_ had an ideal toxicity profile, making it a suitable candidate for the development of antimicrobial drugs.

## 1 Introduction

The emergence and dissemination of antimicrobial-resistant bacteria pose a challenge to the conventional antibiotics used in various clinical practices. This has led to considerable public health concerns and demands for alternative treatments (Ezat et al., 2014). Along with this, more than 80% of microbial infections are generally biofilm-based. Biofilms are structural communities encased in a self-secreted exopolymorphic substance that have shown 10,000 times more resistance to conventional antibiotics (Davies, 2003; Caraher et al., 2007). Hence, it has become imperative to explore new alternatives to inhibit biofilms with nonconventional drugs.

In recent years, the interest and demand for medicinal plants to cure various diseases have increased, as they show negligible negative effects, better acceptability, and an inexpensive nature (Pal and Shukla, 2003). Various bioactive compounds present in medicinal plants can interact in harmony with each other and with the target enzymes or proteins of the microbes. This helps decrease possible side effects caused by their use and also aids in enhancing the immune system (Rasool, 2012; Sarkar et al., 2023). Hence, it is worth exploring the biological potential of bioactive compounds isolated from plant resources. However, one major limitation and challenge associated with contemporary ethnopharmacological research is that plant extracts have a very complex nature. They are composed of many bioactive compounds with diverse structures and biological potential, and some of them may constitute a confounder in the overall activity of plant extracts. However, the discovery of new analytical chromatographic and spectroscopic techniques and computer-aided drug screening methods has revolutionized ethno-pharmacological studies (Ononamadu and Ibrahim, 2021).

Structural entities with cyclohexanoid cores rich in hydroxyl groups obtained from natural sources exhibit much biological potential. Quinic acid ((1R,3R,4R,5R)-1,3,4,5-tetrahydroxycyclohexane-1-carboxylic acid) (QA), a cyclohexanecarboxylic acid, is found in several plants like *Achillea pseudoaleppica*, *Citrus reticulata, Coffea arabica*, *Phagnalon saxatile* subsp. Saxatile, *Rumex nepalensis, Haematocarpus validus*, *Hypericum empetrifolium*, and *Ziziphus lotus* L (Benali et al., 2022). In addition, it is one of the bioactive components in macrofungi (*Coprinus comatus*) (Karaman et al., 2021).

QA is generally present in free form or as esters in various plants. It has also been recognized as an important biogenetic precursor for the biosynthesis of aromatic natural products through the shikimate pathway (Kramer et al., 2003; Arya et al., 2014). In addition, QA has also been found to show several therapeutic properties as an antimicrobial, antifungal, cytotoxic, antidiabetic, insecticidal, anticancer, antioxidant, and analgesic agent (Pero and Lund, 2009; Bai et al., 2018; Liu et al., 2020; Karaman et al., 2021; Samimi et al., 2021).

Various researchers have explored the antibacterial potential and antibiofilm activity of quinic acid (Chang et al., 2018; Lu et al., 2021). However, there are few reports regarding the testing of the antibacterial potential of quinic acid derivatives (Gohari et al., 2010) or their synergistic interaction with standard antibiotics, antibiofilm activity, toxicity analysis, and drug-likeness properties. Moreover, in our study, quinic acid has been isolated from dropped citrus fruits that are generally regarded as waste. Nowadays, the recovery of various biologically active compounds from fruit and vegetable waste has gained attention as a green approach to recovering valuable compounds with nutraceutical potential (Nirmal et al., 2023). Along with this, the discovery of new drugs is a challenging and burdensome process as it is associated with various methodological complexities and limitations. The discovery of computer-aided drug evaluation methods like molecular docking studies and ADMET analysis has revolutionized the discovery process and justifies the novelty of our work (Pinz and Rastelli, 2019; Vardhan and Sahoo, 2020).

The current study is a continuation of our previous communication about the ethyl acetate fraction of the methanol extract of *C. reticulata* Blanco fruits dropped in April that exhibited maximum antibacterial potential (Heena et al., 2022). Citrus fruits show enormous fruit drop starting from fruit set till harvesting, leading to huge fruit loss, environmental pollution, and economic loss worldwide; therefore, any method that could make use of dropped fruit merits considerable attention (Thind and Kumar, 2008).

In the present investigation, QA, a major compound in the ethyl acetate fraction of methanol extract of fruits dropped in April, was isolated, derivatized, and tested for antibacterial potential. The investigation was further elaborated by the study of QA’s synergistic interaction with standard antibiotics; the antibiofilm activity of the most effective treatment; molecular docking studies of QA and its derivatives to predict their binding affinity with DNA gyrase, transpeptidase, and β-lactamase; and a determination of their toxicity analysis and drug-like properties through absorption, distribution, metabolism, excretion, and toxicity (ADMET) analysis.

The use of molecular docking techniques is increasing due to the synergistic connection between medicinal chemistry and molecular simulation and bioinformation (Okada et al., 2010; Ramirez, 2016). Along with these computational tools, ADMET analysis has become a widely used method of biological research to study the toxicity profile and drug-likeness of various drug candidates**.**

## 2 Materials and methods

The preparation of methanol extract and its fractions, along with testing of their antibacterial potential, was done according to the method given by Heena et al. (2022). The ethyl acetate fraction revealing maximum antibacterial potential was further characterized by gas chromatography/mass spectrometry (GC-MS) analysis (Supplementary Figure S1, S2) according to the method reported by Heena et al. (2022).

### 2.1 Isolation of quinic acid from ethyl acetate fraction

The ethyl acetate fraction (8 g) containing QA as a major compound was chromatographed over silica gel (300 g) using H_2_O:methanol as a solvent system of increasing polarity. The H_2_O:methanol fractions (30:70) and (20:80) showed the presence of three compounds having similar Rf values. These fractions were mixed (3.95 g) and further subjected to column chromatography using silica gel (200 g) and butanol:acetic acid:water (B:A:W) as a solvent system of increasing polarity (El-Bassossy, 2022) (Supplementary Table S1, S2). QA was isolated in pure form in two fractions of B:A:W, 3:1:1 and 4:1:0, and its structure was confirmed using spectroscopic techniques.

### 2.2 Derivatization of QA

#### 2.2.1 Quinic acid to 3,4-O-isopropylidenequinic acid 1,5-lactone (QA_1_)

**FIGURE 1:**
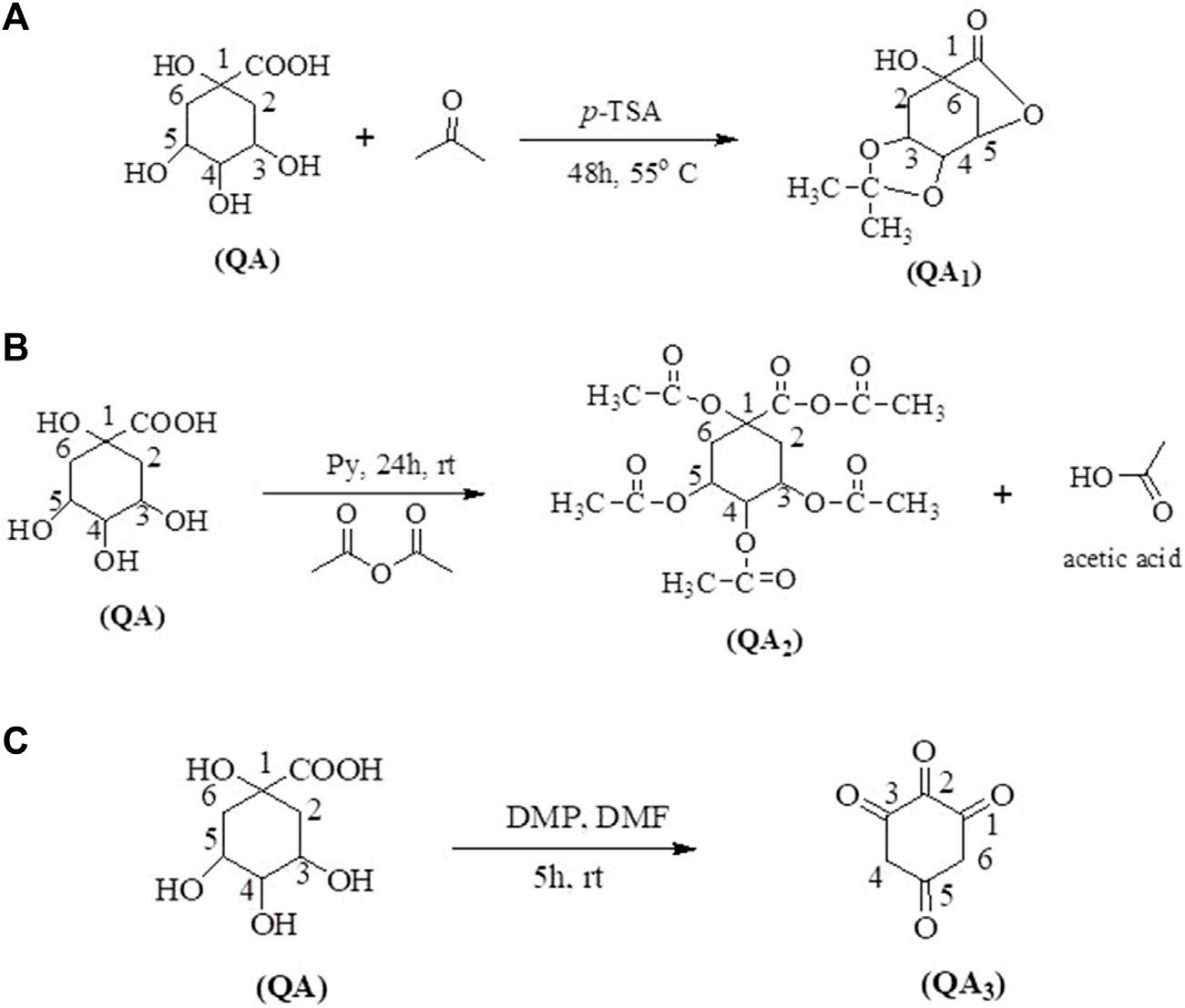
Synthetic routes of target compounds; QA_1_ **(A)**, QA_2_ **(B)**, and QA_3_ **(C)** from QA.

QA (5 mmol, 1.00 g) was mixed with acetone (5 mL) and *p*-toulenesulfonic acid (*p*-TSA) (5 mmol; 0.95 g) as an acidic catalyst. The reaction mixture was refluxed at 55°C for 48 h. The reaction mixture was then diluted with dichloromethane (DCM) and brine solution (10% NaCl) in a separatory funnel to remove impurities (Figure 1A). The compound formed was collected from the DCM layer, which was further evaporated, leaving behind 3,4-o-isopropylidenequinic acid 1,5-lactone (QA_1_) as a final product (Wang et al., 2013).

#### 2.2.2 Quinic acid to 1,3,4,5-tetraacetoxycyclohexylaceticanhydride (QA_2_)

QA (5 mmol; 1.00 g) was dissolved in acetic acid (95% in H_2_SO_4_) and added to pyridine–acetic anhydride (1:1; 10 mL). The mixture was stirred for 24 h at room temperature. The solvent was evaporated with a rotary evaporator. The compound left behind was purified in the separatory funnel using ethyl acetate and water (1:1) containing sodium bicarbonate (20% in distilled water) (Figure 1B). The pure compound was collected in the ethyl acetate layer.

#### 2.2.3 Quinic acid to cyclohexane-1,2,3,5-tetraone (QA_3_)

In a 50-mL round bottom flask, QA (5 mmol; 1.00 g) was dissolved in DMF (10 mL) and 5.00 equiv. of Dess–Martin periodinane (DMP) was added. The reaction mixture was stirred at 25°C for 5 h. The reaction was further quenched with the addition of a 1:1 mixture of a saturated solution of NaHCO_3_:Na_2_S_2_O_3_ (20 mL) and stirred for 25 min. The aqueous layer of the reaction mixture was extracted with ethyl acetate (3 × 10 mL), saturated NaHCO_3_ (3 × 10 mL), and brine solution (2 × 10 mL) and further dried over Na_2_SO_4_. The solvent was removed with the help of a rotary evaporator under vacuum. The crude product was purified using column chromatography (Sanichar et al., 2018) (Figure 1C).

The physical properties and spectroscopic data of the target compounds (QA, QA_1_, QA_2_, and QA3) are listed as follows.

**QA:** mp:160–162°C, yield 27.84%, FT-IR band (cm^−1^): 1,133 cm^−1^, 1,267 cm^−1^, 1,449 cm^−1^, 1,677 cm^−1^, 2,929 cm^−1^, 3,332 cm^−1^, and 3,507 cm^−1^,^1^HNMR signal (400 MHz, DMSO) (δ in ppm): 2.80–2.84 (m, 4H), 3.20–3.70 (m, 3H), 4.55 (s, 3H), 5.33 (s, 1H), 12.20 (s, 1H), ^13^CNMR signal (100 MHz, DMSO) (δ in ppm): 39.84, 40.05, 67.20, 69.47, 74.89, 176.12 (Supplementary Figure S3–S5).

**QA**_**1**_ **:** mp:168–170°C, yield 42%, FT-IR band (cm^−1^): 3,503 cm^−1^, 2,929 cm^−1^, 2,866 cm^−1^, 1707 cm^−1^, 1,658 cm^−1^, 1,438 cm^−1^, and 1,222 cm^−1^, ^1^HNMR signal (400 MHz, CDCl_3_) (δ in ppm): 1.46 (s, 3H), 1.99 (s, 3H), 2.07 (s, 1H), 2.08–2.10 (m, 2H), 2.13–2.16 (m, 2H), 2.35–2.42 (m, 1H), 2.54–2.59 (m, 1H), 2.63–2.68 (m, 1H), ^13^CNMR signal (100 MHz, CDCl_3_) (δ in ppm): 27.01, 32.5, 40.58, 63.91, 76.94, 77.26, 77.58, 210.2 (Supplementary Figure S6–S8).

**QA**_**2**_ **:** mp: 170–173°C, yield 58.5%, FT-IR band (cm^−1^): 700 cm^−1^, 752 cm^−1^, 1,066 cm^−1^, 1,129 cm^−1^, 1,215 cm^−1^, 1,263 cm^−1^, 1,367 cm^−1^, 1,438 cm^−1^, 1,487 cm^−1^, 1707 cm^−1^, and 1889 cm^−1^, ^1^HNMR signal (400 MHz, CDCl_3_) (δ in ppm): 1.90–2.05 (m, 6H), 2.06–2.09 (m, 4H), 2.10–2.60 (m, 9H), 5.03–5.53 (m, 3H), ^13^CNMR signal (100 MHz, CDCl_3_) (δ in ppm): 20.51, 32.52, 64.05, 78.0, 82.0, 168.0, 172.0 (Supplementary Figure S9–S11).

**QA**_**3**_ **:** mp: 120–124°C, yield 32.02%, FT-IR band (cm^−1^): 1,438.7 cm^−1^, 1,494 cm^−1^, and 1,647 cm^−1^, ^1^HNMR signal (400 MHz, DMSO) (δ in ppm): 2.8 (s, 4H), ^13^CNMR signal (100 MHz, DMSO) (δ in ppm): 40.58, 190.73, 191.25, 203.91 (Supplementary Figure S12–S14).

### 2.3 Antibacterial activity

#### 2.3.1 Antibacterial potential of QA and its derivatives

The antibacterial activity of QA and its derivatives against *Escherichia coli* MTCC 443, *Yersinia enterocolitica* MTCC 859, *Staphylococcus aureus* MTCC 96, and *Bacillus* sp. MTCC 441 was determined by the disk diffusion method. The nutrient agar media was prepared, autoclaved, and poured into the sterilized Petri plates (90 mm, HiMedia, India). The media was allowed to gel. A known quantity (50 µL) of the fresh broth cultured bacterial cell (24–48-h-old culture) suspension was spread-plated and allowed to air-dry in a laminar air flow bench (Horizontal, Micro flow, Pvt. Ltd. India) after covering the media with the Petri lid. Stock solutions (2000 μg/mL) of each treatment were prepared in dimethyl sulfoxide (DMSO: 10%) to make various dilutions (100–1,500 μg/mL). A filter paper disk (5–6 mm diameter) was impregnated with 20 µL of a known concentration of each treatment and was shade-dried. These disks were then placed on the agar plate. The plates were incubated at an appropriate temperature (37°C ± 2°C) in a BOD incubator (Remi Lab. Incubator, Ludhiana, India). The filter paper discs containing DMSO (10%) served as control, while streptomycin (10 μg/mL) was used as the standard antibiotic. After incubating the plates, the diameters of the zone of inhibition (mm) were measured (De Zoysa et al., 2019).

#### 2.3.2 Determination of minimum inhibitory concentration (MIC)

The MIC was determined by broth macro-dilution assay (Ghatage et al., 2014). This method served as a modified micro-dilution assay, as reported in the literature (Vu et al., 2017). Two- or ten-fold dilutions of the compounds in nutrient broth (2–5 mL) in test tubes were obtained. The bacterial inoculum (0.5 McFarland diluted 1/150 in broth) was transferred in each tube, and the inoculated tubes were incubated at optimum temperature for 24 h. The positive control included the antibiotic streptomycin, which served as the standard. The MIC was taken at the least concentration of extracts that inhibited the detectable growth of the tested bacteria.

### 2.4 Bacterial cell constituent release

The release of bacterial cell constituents into the supernatant was tested according to the reported method (Lv et al., 2011). The cells from the bacterial cultures (100 mL) were collected by centrifugation at 5,000 rpm for 10 min. They were washed three times with double-distilled water and resuspended in phosphate buffer saline (0.1 M, pH 7.0). Then, the bacterial cell suspensions (100 mL) were incubated at 37°C for 1 h in the presence of each treatment at its MIC value. The samples (2 mL) were centrifuged at 12,000 rpm for 2 min, and the supernatant (1 mL) obtained in each case was studied for absorption under a UV–visible spectrophotometer (Shimadzu) at 260 nm to determine the release of bacterial cellular contents. A correction was made for the absorption of the suspension with phosphate-buffered saline (PBS; pH 7.0) containing the same concentration of compounds after 2 min of contact with the tested bacterial strains along with the untreated cells taken as the control.

### 2.5 Evaluation of the synergistic interaction

The synergistic interaction of QA and the most effective derivative with the standard antibiotic streptomycin was determined using the checkerboard method (Pourkhosravani et al., 2021). On a 96-well microtiter plate, each treatment was diluted in the well columns, and the standard antibiotic was diluted in rows at two-fold dilution of MIC ranging from MIC to 1/16 of MIC. Briefly, each treatment (50 µL) at MIC was mixed with streptomycin (50 µL) at MIC with serial two-fold dilution in Muller–Hinton broth. After that, the log-phase bacterial (100 µL) inoculum was added to all the wells and incubated at 37°C for 24 h. The MICs of each treatment and its combinations were recorded. The results were expressed in the fractional inhibitory concentration index (FICI) as follows:

$$\text{FICI}={\text{FIC}}_{A}+{\text{FIC}}_{B}$$

FIC_A_ = MIC of treatment in combination/MIC of treatment alone.

FIC_B_ = MIC of streptomycin in combination/MIC of streptomycin alone.

FICI ≤0.5 shows synergistic interaction; 0.5 < FICI ≤1 shows an additive effect; 1 < FICI ≤4 reveals indifference or no effect; and FICI ≥4 shows antagonistic effects (Gutierrez et al., 2009).

### 2.6 Antibiofilm activity

The antibiofilm activity of the most effective treatment was studied using the crystal violet method (Sanchez et al., 2016) to check the mechanism of antibacterial activity by estimating the specific biofilm formation (SBF) through UV spectroscopy.

The nutrient broth (5 mL) was added to a 96-well microtiter plate along with 50 μL of OD_560_ = 0.02 (10^6^ CFU/mL) microbial culture followed by the addition of the most effective treatment at its MIC, and 25%, 50%, and 75% of the MIC value. The plate containing streptomycin served as the standard, while the one without treatment served as the control. The plates were further incubated at 37°C for 48 h without disturbance, allowing for ring formation. After incubation, the supernatant was removed very carefully with the help of a micro-pipette, and each well was washed thoroughly with distilled water (three times) to remove planktonic or unadhered cells. The plates were then dried in air for half an hour. Thereafter, 5 mL of crystal violet dye (1% aqueous solution) was added to the plates and kept at room temperature for 15 min. After that, the solution was discarded, followed by gentle washing with distilled water (three times) to remove the excess stain. Then, 200 μL of ethanol (95%) solution was added to each well and incubated for 15 min. After that, the absorbance was recorded at 570 nm using an ELISA plate reader (Thermo Scientific, Multiscan Go). The specific biofilm formation was calculated as follows:

$$\text{SBF}=\left(\text{AB}-\text{ CW}\right)/G,$$

where SBF is the specific biofilm formation.

AB is the OD at 570 nm of the attached and stained bacteria.

CW is the OD at 570 nm of the stained control wells containing only the bacteria-free medium.

G is the OD at 630 nm of cell growth in broth (Niu and Gilbert, 2004).

The SBF values were classified into three categories.

Strong biofilm: SBF> 2.00.

Intermediate biofilm: SBF between 1 and 2.

Weak biofilm SBF <1.00.

### 2.7 *In silico* studies

#### 2.7.1 Molecular docking of QA and its derivatives against targeted proteins

The crystal structures of the target enzyme*,* that is, the DNA gyrase of the bacterial strains (PDB id: 1KZN), transpeptidase (PDB id: 1MWT), and β-lactamase (PDB id: 6BU3), were obtained from the Protein Data Bank (Lafitte et al., 2002). The structure of the receptors, as well as the structures of the four ligands (QA, QA_1_, QA_2_, and QA_3_), were processed using AutodockTools (Morris et al., 2009). For protein preparation, all water molecules, the cognate ligands, clorobiocin, streptomycin, and ampicillin were removed from the PDB file, and docking grid boxes of dimensions 88 Å × 92 Å × 86 Å (scaling factor: 0.510 Å), 68 Å × 58 Å × 126 Å (scaling factor: 0.999 Å), and 104 Å × 70 Å × 108 Å (scaling factor: 0.525 Å) were defined over selected chain A of DNA gyrase, transpeptidase, and β-lactamase, respectively, to accommodate the whole target enzymes. The ligands were prepared by defining the root of each ligand and assigning the Gasteiger charges (Gasteiger and Marsili, 1980). To expedite the docking studies while maintaining accuracy, AutoDock-GPU4 (v1.5) (Santos-Martins et al., 2021), an OpenCL implementation of the widely used AutoDock4 (Morris et al., 2009), was employed. Specifically, we used a docking protocol consisting of 250 Lamarckian genetic algorithm (LGA) (Morris et al., 1998) runs for the global search over a population of 300 individuals. Furthermore, 1,000 iterations of the Solis–Wets local search algorithm (Solis and Wets, 1981) were included, which allowed up to 25 million energy evaluations along with a 100% local search rate. Furthermore, to confirm the robustness of our docking protocol, a cognate ligand (clorobiocin) was also docked. Structural superposition was performed for the crystallized and docked conformations of clorobiocin, transpeptidase, and β-lactamase using PyMol. The analysis and visualization of the protein–ligand interactions were carried out using ChimeraX and Discovery Studio (Pettersen et al., 2021).

#### 2.7.2 Toxicity, physiochemical properties, and ADMET analysis

The toxicity analysis of compounds was determined from ProTox-II analysis. Class 1 compounds are highly toxic and become less toxic toward class 6 toxicity, which can be considered safe molecules (Drwal et al., 2014). The pharmacokinetic properties of QA and its derivatives were studied by ADMET analysis, which provides an insight into the molecules and helps identify their drug-like potential (Chandrasekaran et al., 2018). In the present study, the physiochemical properties (Lipinski parameters) and the ADMET profiles of QA and its derivatives were evaluated by the pkCSM web facility (http://structure.bioc.cam.ac.uk/pkcsm) using the provided SMILE string of the molecules (Li, 2001).

### 2.8 Statistical analysis

All the results were expressed in terms of mean ± standard deviation with three replications. Two-way ANOVA followed by Tukey’s B test was carried out for the investigation of antibacterial activity data, and *p* < 0.05 was accepted as significant.

## 3 Results and discussion

### 3.1 Chemistry

QA was found to be the major compound present in the ethyl acetate fraction of the methanol extract of citrus fruits dropped in April, as revealed by GC-MS analysis (Supplementary Figure S1, S2). QA has also been reported to be present in the dropped immature fruits of different citrus species during the early stages of development (Albertini et al., 2006; Marrubini et al., 2015). Various chlorogenic acids (esters of caffeic acid and quinic acid) have been isolated from the ethyl acetate extract of *Zanthoxylum bungeanum* leaves (Chang et al., 2018). It was isolated from the ethyl acetate fraction by column chromatography as a white solid with a melting point in the range of 160–162°C. It was derivatized into QA_1_, QA_2_, and QA3. The structures of QA and its derivatives were confirmed using spectroscopic techniques (Supplementary Figure S3–S14) and by reference to the published data in the literature (Wang et al., 2013).

### 3.2 Antibacterial activity of QA and its derivatives

**FIGURE 2:**
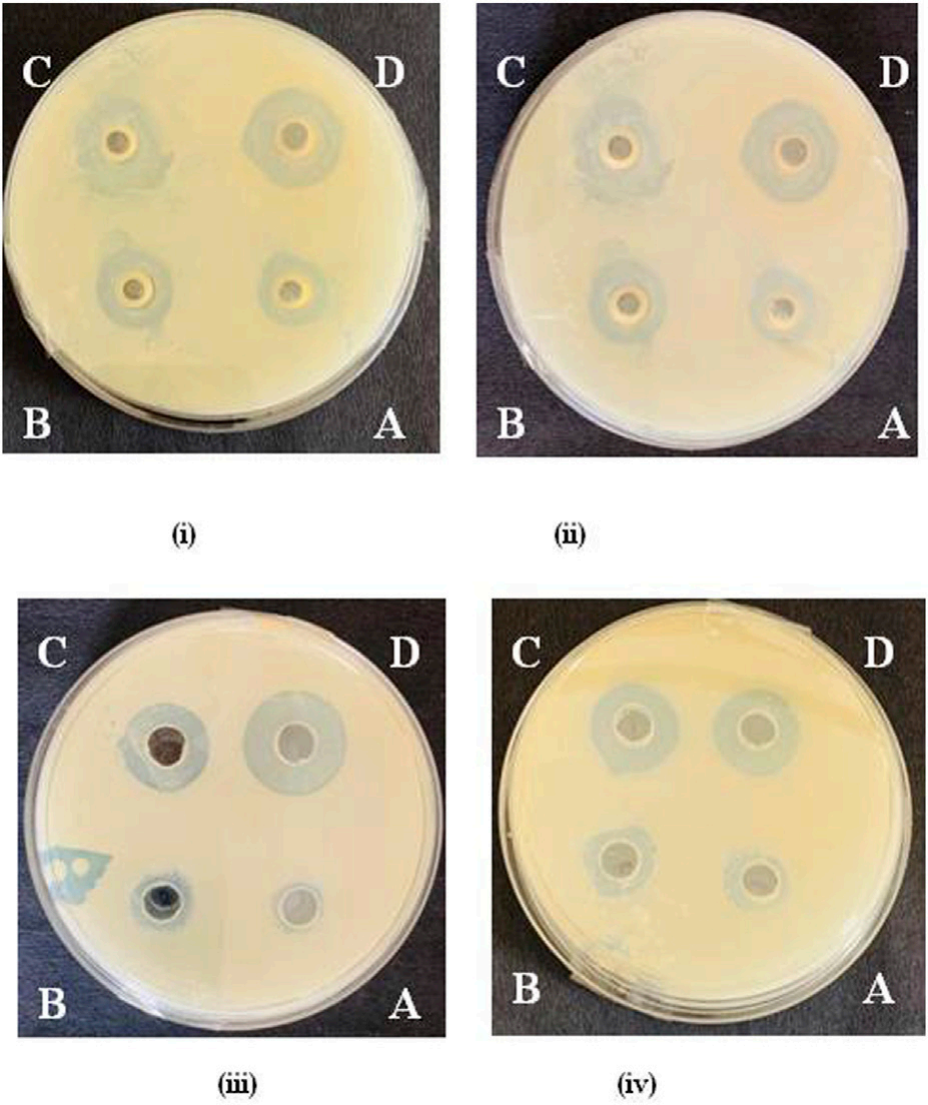
Zone of inhibition by QA_1_ against *E. coli* (i), *Bacillus* spp. (ii), *Staphylococcus aureus*= (iii), and *Y. enterocolitica* (iv) @ 250 μg/mL (A), 500 μg/mL, (B), 1,000 μg/mL (C), and 1,500 μg/mL (D).

Among QA and its derivatives, the compound QA_1_ showed maximum antibacterial potential against all the tested bacteria with maximum diameter inhibition zones at 1,500 μg/mL of 33.10 mm, 38.86 mm, 32.13 mm, and 35.46 mm against *E. coli*, *Bacillus sp*., *Y. enterocolitica,* and *S. aureus,* respectively (Figure 2). This was followed by compound QA_2_, which showed 29.30 mm and 22.50 mm zones of inhibition against *Bacillus sp*. and *S. aureus*, respectively, and compound QA_3_, which showed zones of inhibition of 27.20 mm and 24.61 mm against *E. coli* and *Y. enterocolitica*, respectively, at the same concentration, as shown in Supplementary Tables S3–S6.

**FIGURE 3:**
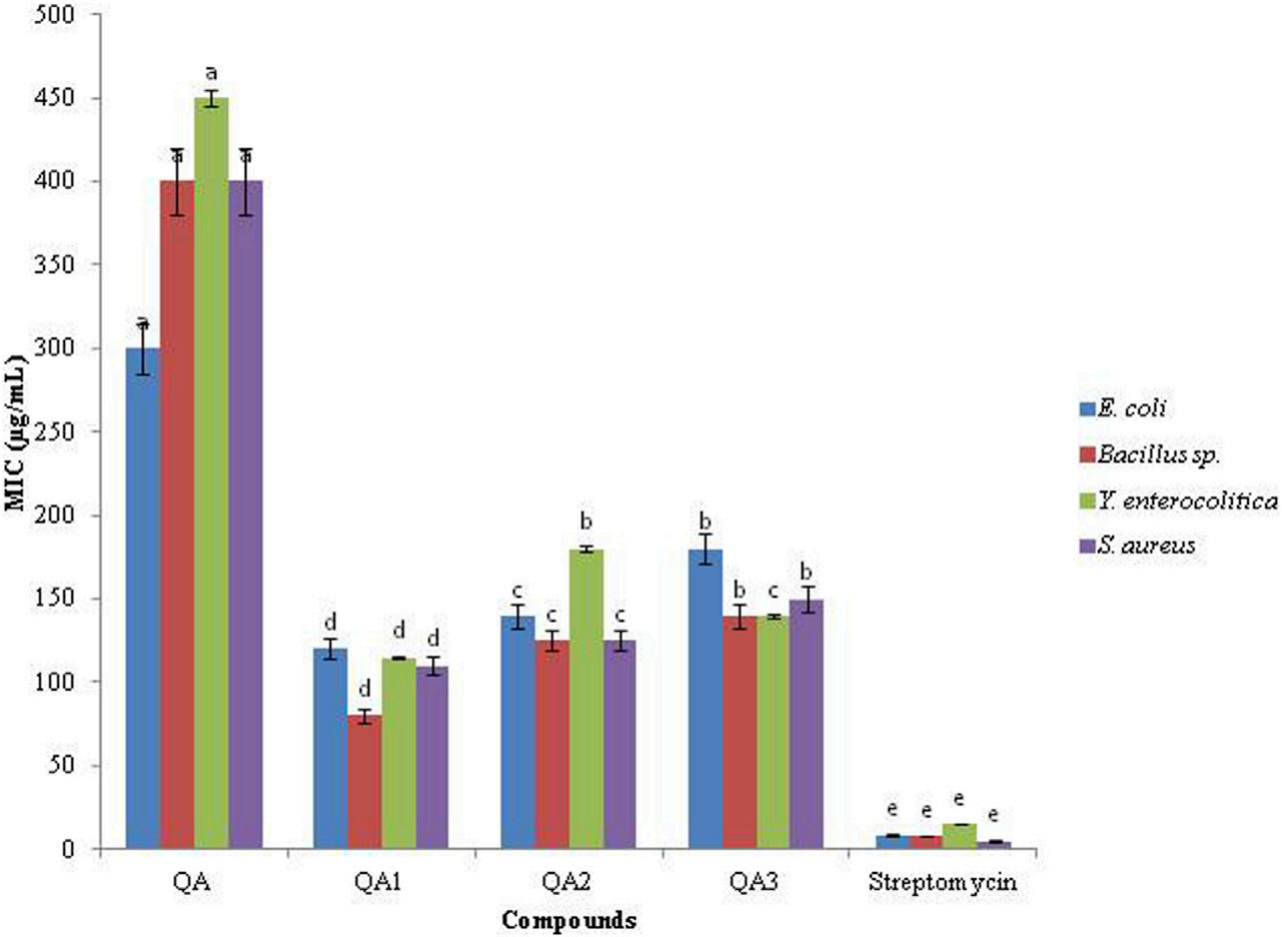
MIC values (µg/mL) of compounds.

The compound QA_1_ also exhibited the lowest MIC values against all the tested bacteria in the range of 80–120 μg/mL (Figure 3). QA was found to be the least effective against all the bacterial strains, with maximum values of MIC (300–450 μg/mL) against the tested bacteria.

Based on their MIC values, the antibacterial activity of QA and its derivatives against the tested bacteria followed the following order:

Streptomycin > QA_1_ > QA_2_ > QA_3_> QA (*Bacillus sp*. and *S. aureus*).

Streptomycin > QA_1_ > QA_3_> QA_2_ > QA (*E. coli* and *Y. enterocolitica*).

Depending upon the MIC values, the antibacterial potential of QA_1_ (most effective treatment) against the tested bacteria decreased as follows.

*Bacillus sp*. > *S. aureus* > *Y. enterocolitica* > *E. coli*.

All the treatments were found to be less effective than the standard streptomycin, which showed an MIC in the range of 5–15 μg/mL against all the tested bacterial strains. All the results were significantly different from each other by (*p* < 0.05), as indicated by two-way ANOVA followed by Tukey’s-B test.

QA has been found effective against several Gram-positive and Gram-negative bacteria (Ercan and Dogru, 2022). It has been reported in the literature that QA inhibited the growth of *S. aureus* ATCC 6538 by reducing the bacterial DNA content, having an MIC of 2.5 mg/mL (Bai et al., 2018). Various QA derivatives isolated from *Z. bungeanum* leaves via ethyl acetate extraction exhibited consistent and moderate bactericidal activity with an MIC and minimum bactericidal concentration (MBC) of 5 mg/mL and 10 mg/mL, respectively (Chang et al., 2018). Various acyl quinic acids detected in *Geigeria alata* extracts exhibited antibacterial potential against *S. aureus* in which 3,4,5-tricaffeoylquinic acid possessed maximum antibacterial activity (MIC/MBC = 2.5 mg/mL) (Zheleva-Dimitrova et al., 2017).

In our study, QA_1_ carrying a lactone moiety has been found to be more effective than its parent compound, QA. Similar results have also been reported in the literature where the antibacterial potential of QA was less than that of its derivative, QA_1_. QA showed MIC values at 100 μg/mL, 1,024 μg/mL, 1,024 μg/mL, and 1,021 μg/mL; however, QA_1_ showed comparatively lower MIC values at 100 μg/mL, 512 μg/mL, 512 μg/mL, and 512 μg/mL against *S. aureus*, *Staphylococcus epidermidis*, *Pseudomonas aeruginosa,* and *Mycobacterium tuberculosis,* respectively (Rezende et al., 2014). It has been reported that QA alters the fluidity of cell membranes by disturbing oxidative phosphorylation along with altering levels of fatty acids and glycerophospholipids. After crossing the cell membrane, it inhibits protein synthesis by altering ribosome function and synthesis of aminoacyl-tRNAs (Bai et al., 2019).

### 3.3 Cell constituent release

**FIGURE 4:**
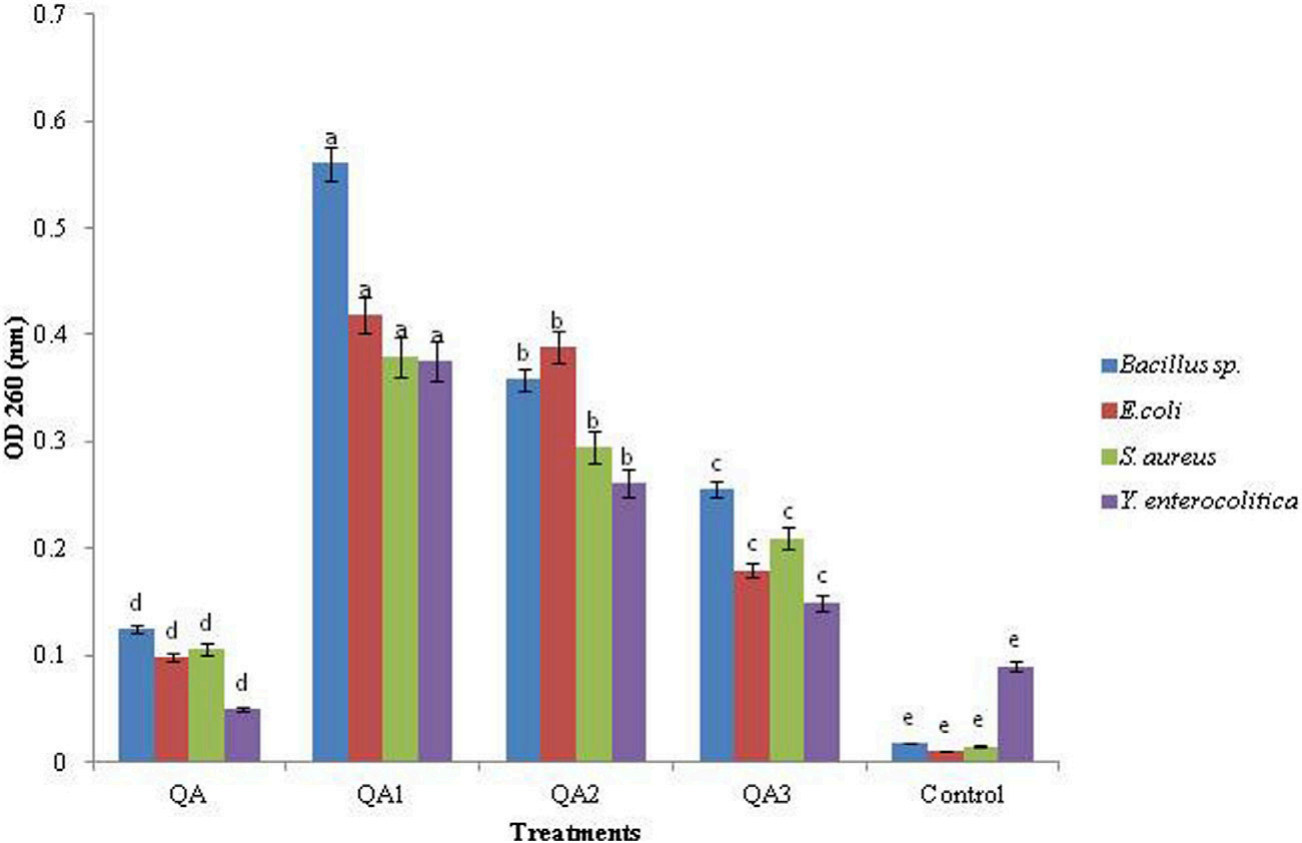
Effect of QA and derivatives on the leakage of cellular components in the tested bacterial strains.

All the compounds caused a significant increase in cell constituent release in all the tested bacteria compared to the control, as indicated by an increase in OD values. Among the four compounds, the maximum cell constituent release was caused by QA_1_, followed by QA_2_, QA_3_, and QA. Among the four tested bacteria, QA_1_ caused the maximum cell constituent release in *Bacillus* sp., having the highest OD value, 0.56 ± 0.80, followed by *E. coli* (OD; 0.419 ± 0.23), *S. aureus* (OD; 0.380 ± 0.30), and *Y. enterocolitica* (OD; 0.375 ± 0.96), as shown in Figure 4 *.* On the other hand, QA_2_ caused the maximum release of cell constituents in *E coli* (OD; 0.389 ± 0.64), followed by *Bacillus* sp. (OD; 0.358 ± 0.51), *S. aureus* (OD; 0.295 ± 0.45), and *Y. enterocolitica* (OD; 0.261 ± 0.55)*.* QA_3_ showed the maximum release of cell constituents in *Bacillus* sp (OD; 0.256 ± 0.72), followed by *S. aureus* (OD; 0.210 ± 0.84), *E. coli* (OD; 0.180 ± 0.72), and *Y. enterocolitica* (OD; 0.150 ± 0.50). QA showed the lowest bacterial constituent release in *Bacillus* sp (OD; 0.125 ± 0.22), followed by *S. aureus* (OD; 0.106 ± 0.23), *E. coli* (OD; 0.098 ± 0.46), and *Y. enterocolitica* (OD; 0.050 ± 0.65) (Figure 4). These differences in cell constituent releases in four tested bacteria may be due to differences in the chemical structures of the tested compounds. Loss of bacterial cell constituents means that the treatment resulted in irreversible damage to the cytoplasmic membrane. It has been reported in the literature that various organic acids strongly affected bacterial cell membrane integrity, as indicated by an increase in OD_260_ values and bacterial liquid conductivity (Liu et al., 2020).

Our results were also supported by the fact that QA had a damaging effect on the cell membrane as observed by transmission electron microscopy, which led to hyperpolarization and decreased membrane fluidity by interacting with the phenylalanine residues of the membrane protein as observed by fluorescence quenching technique (Bai et al., 2018).

### 3.4 Synergistic potential

Synergistic interaction between plant-derived natural products and synthetic antibiotics may allow the use of a lower doses of antibiotics to minimize their side effects (Nidhi et al., 2020). The synergistic interactions of the parent compound (QA) and the most effective derivative (QA_1_) against a standard antibiotic, streptomycin, were studied through the checkerboard method.

The results were expressed in terms of FICI, as shown in Table 1. Among all the treatments, the compound QA_1_ carrying a lactone moiety showed synergistic interaction with streptomycin against all the tested bacteria, *E. coli, Y. enterocolitica*, *Bacillus* spp. and *S. aureus* with FICI values of 0.31, 0.37, 0.37, and 0.29 respectively. QA showed indifferent behavior with streptomycin against *E. coli*, *Y. enterocolitica,* and *S. aureus* with FICI values at 1.12, 1.00, and 1.12, respectively, while it showed additive behavior with streptomycin against *Bacillus* sp. with an FICI value of 0.62, as shown in Table 1. QA_1_ showed synergistic behavior and a reduced MIC of the standard antibiotic (streptomycin) from 8.70 to 0.54 μg/mL against *E. coli,* from 8.0 to 1.0 μg/mL against *Y. enterocolitica,* from 15.0 to 1.80 μg/mL against *Bacillus* sp., and from 5.0 to 0.62 μg/mL against *S. aureus*.

**TABLE 1:** Synergistic interaction of QA and QA_1_ with streptomycin: standard antibiotic.

Bacteria	Related indexes	Compound QA_1_	Streptomycin	Compound QA	Streptomycin
***E. coli***	MIC_a_	120.0	8.70	350.0	8.70
	MIC_c_	30.0 (MIC/4)	0.54 (MIC/16)	350.0 (MIC)	0.54 (MIC/16)
	FIC	0.25	0.06	1.0	0.06
	**FICI**	**0.31^b^ (S)**		**1.06^b^ (I)**	
***Y. enterocilitia***	MIC_a_	115.0	8.0	450	8.0
	MIC_c_	28.75 (MIC/4)	1.0 (MIC/8)	450 (MIC)	1.0 (MIC/8)
	FIC	0.25	0.12	1.00	0.12
	**FICI**	**0.37^a^ (S)**		**1.12^a^ (I)**	
***Bacillus* sp**	MIC_a_	80.0 (MIC/4)	15.0 (MIC/8)	400.0 (MIC/2)	15.0 (MIC/8)
	MIC_c_	20.0	1.80	70.0	1.87
	FIC	0.25	0.12	0.50	0.12
	**FICI**	**0.37^a^ (S)**		**0.62^c^ (A)**	
***S. aureus***	MIC_a_	110	5.0	400.0	5.0
	MIC_c_	18.75 (MIC/6)	0.62 (MIC/8)	200.0 (MIC/2)	0.31 (MIC/8)
	FIC	0.17	0.12	0.50	0.62
	**FICI**	**0.29^c^ (S)**		**1.12^a^ (I)**	

MIC_a_: MIC of the sample tested alone (µg/mL), MIC_c_: MIC of a combination of treatment with streptomycin (µg/mL), FIC: MIC of the combination/MIC alone; FICI, sum of the FIC of the compound and the FIC of streptomycin.

Results are interpreted as synergy (S, FICI ≤0.5), additive (A, 0.5 <FICI≤1), indifferent (I, 1<FICI≤4), or antagonism (AN, FICI>4). The mean FICI values shown in the table above followed with different superscripts are significantly different (*p* < 0.05) using two-way ANOVA, followed by Tukey’s-B test.

QA_1_, when used in combination with streptomycin, resulted in an 8–16× reduction in the MIC values of streptomycin. Our results are supported by the literature where sesquiterpene lactones were isolated from *Centratherum punctatum Cass*. showed limited antibacterial activity individually against drug-resistant *E. coli* and *K. pneumoniae*. However, when combined with ampicillin, they exhibited a synergistic effect, significantly enhancing antibacterial efficacy. The MIC values of the standard antibiotic, ampicillin, when used with different lactones, showed a reduction from 1,250 μg/mL to 78–625 μg/mL for *E. coli* and from 2,500 μg/mL to 78–1,250 μg/mL for *K. pneumoniae*. The experiment illustrated a pronounced synergistic interaction, as indicated by the FICI index values ranging from 0.185 to 1.00 for *E. coli* and 0.28 to 0.75 for *K. pneumoniae* (Chukwujekwu et al., 2018). The bactericidal activity of caffeic acid was improved when quinic acid was used in synergy due to a reduction in log survival ratio (3.71 ± 0.23 CFUs/mL vs. 5.45 ± 0.39 CFUs/mL when used alone) at low pH, thereby suggesting the synergistic potential of quinic acid (Kabir et al., 2014). It has also been reported that QA exhibited a synergistic antibiofilm effect with levofloxacin at concentrations below its MIC (Lu et al., 2021).

### 3.5 Antibiofilm activity

Biofilm represents a multicellular entity of the bacterial colonies present in the self-produced matrix that protects bacteria under adverse environmental conditions. It is considered one of the major factors responsible for prolonged bacterial infections as it protects them from antibiotic treatment (Jain and Parihar, 2018). QA_1_ and its combination with the standard antibiotic, streptomycin, were tested for their antibiofilm activity against all the test bacteria as it revealed maximum antibacterial potential against these bacteria along with a synergistic interaction with streptomycin. The results were further interpreted in terms of specific biofilm formation values.

QA_1_, when used alone, exhibited intermediate biofilm formation with SBF values of 1.33, 1.25, and 1.87 against *E. coli*, *Bacillus* sp. and *S. aureus,* respectively, and strong biofilm formation with an SBF value of 2.55 against *Y. enterocolitica*. However, when QA_1_ @ MIC/4 was used in combination with streptomycin (MIC/8), the pair showed weak biofilm formation with SBF values of 0.98 and 0.80 against *Y. enterocolitica* and *Bacillus* sp., respectively. It also showed weak biofilm formation (SBF = 0.90) @ MIC/4 against *E. coli* when used in combination with streptomycin (MIC/16) and intermediate biofilm formation (SBF = 1.20) @ MIC/6 against *S. aureus* when used in combination with streptomycin (MIC/8) (Table 2).

**TABLE 2:** Antibiofilm activity of QA_1_ alone and in combination with a standard antibiotic.

Bacterial strain	SBF				
	Compound QA_1_ (@ MIC values)	Compound QA_1_ + streptomycin	Control	Streptomycin	Tukey mean
***E. coli***	1.33 (I)	0.90 (W)	2.15 (S)	0.83 (W)	1.32^d^
***Y. enterocolitica***	2.55 (S)	0.98 (W)	3.12 (S)	0.67 (W)	1.80^a^
***Bacillus sp***	1.25 (I)	0.80 (W)	2.98 (S)	0.73 (W)	1.46^c^
***S. aureus***	1.87 (I)	1.20 (I)	3.01 (S)	0.54 (W)	1.65^b^
**Tukey mean**	1.76^b^	0.98^c^	2.75^a^	0.68^d^	

Mean values of SBF. The values shown in the table above followed with different superscripts are significantly different (*p* < 0.05) using two-way ANOVA, followed by Tukey’s B test.

In one study, QA showed inhibition of extracellular polymeric material (EPS) secretion in the biofilm of *P. aeruginosa* at some sub-MICs (Lu et al., 2021). Moreover, QA, when treated @ 0.3125–1.25 mg/mL, significantly reduced biofilm formation of *S. aureus* from 55% to 70% as compared to the control. It significantly reduced biofilm mass by decreasing viability and also reduced the metabolic activity of biofilm cells (Bai et al., 2019). Various chlorogenic acids isolated from an ethyl acetate extract of *Z. bungeanum* leaves showed decreased metabolic activity of the cells in the biofilm of *S. aureus* with 31.1%–65.4% inhibition (Chang et al., 2018). Similar results were reported in which a natural product (α-amylase) showed antibiofilm activity against *P. aeruginosa* and *S. aureus* at 200 μg/mL (Lahiri et al., 2021).

### 3.6 Molecular docking results

Molecular docking is a useful approach for discovering new drugs. It is based on searching for drug targets like enzymes or receptors and also bioactive components in natural products (Gupta and Singh, 2012; Sarkar et al., 2022). DNA gyrase is one of the attractive targets for antibacterial drugs; it is involved in bacterial DNA replication and transcription. This enzyme belongs to the topoisomerases, which control topological transitions of DNA and catalyze negative supercoiling of circular DNA by ATP hydrolysis (Collin et al., 2011). The enzyme has also been used as a target for antibacterial drugs as it is essential for all bacteria and is absent in eukaryotes (Heddle and Maxwell, 2002; Kumar et al., 2014). Docking analysis was carried out to better understand the mode of action of bioactive components against the bacterial enzymes transpeptidase and β-lactamase. Transpeptidases catalyze the nucleophilic carbonyl substitution process involved in cross-linking of peptidoglycan in the bacterial cell wall. β-lactamase inactivates betalactam antibiotics by hydrolyzing the peptide bond of the characteristic four-membered beta-lactam ring, rendering the antibiotic ineffective. By understanding the interactions between target enzymes and the bioactive components through docking studies, researchers can design more potent inhibitors to overcome antibiotic resistance and enhance the efficacy of natural antibiotics in clinical use (Elfaky et al., 2020; Shidiki and Vyas, 2022).

Hence, molecular docking studies were performed to predict the interaction of QA and its derivatives with the binding sites of DNA gyrase, transpeptidase, and β-lactamase. The crystal structures of the three enzymes (PDB codes 1KZN, 1MWT, and 6BU3, respectively) were chosen as the protein model for the current study (Jayashree et al., 2010; Bansal et al., 2014; Elfaky et al., 2020).

**FIGURE 5:**
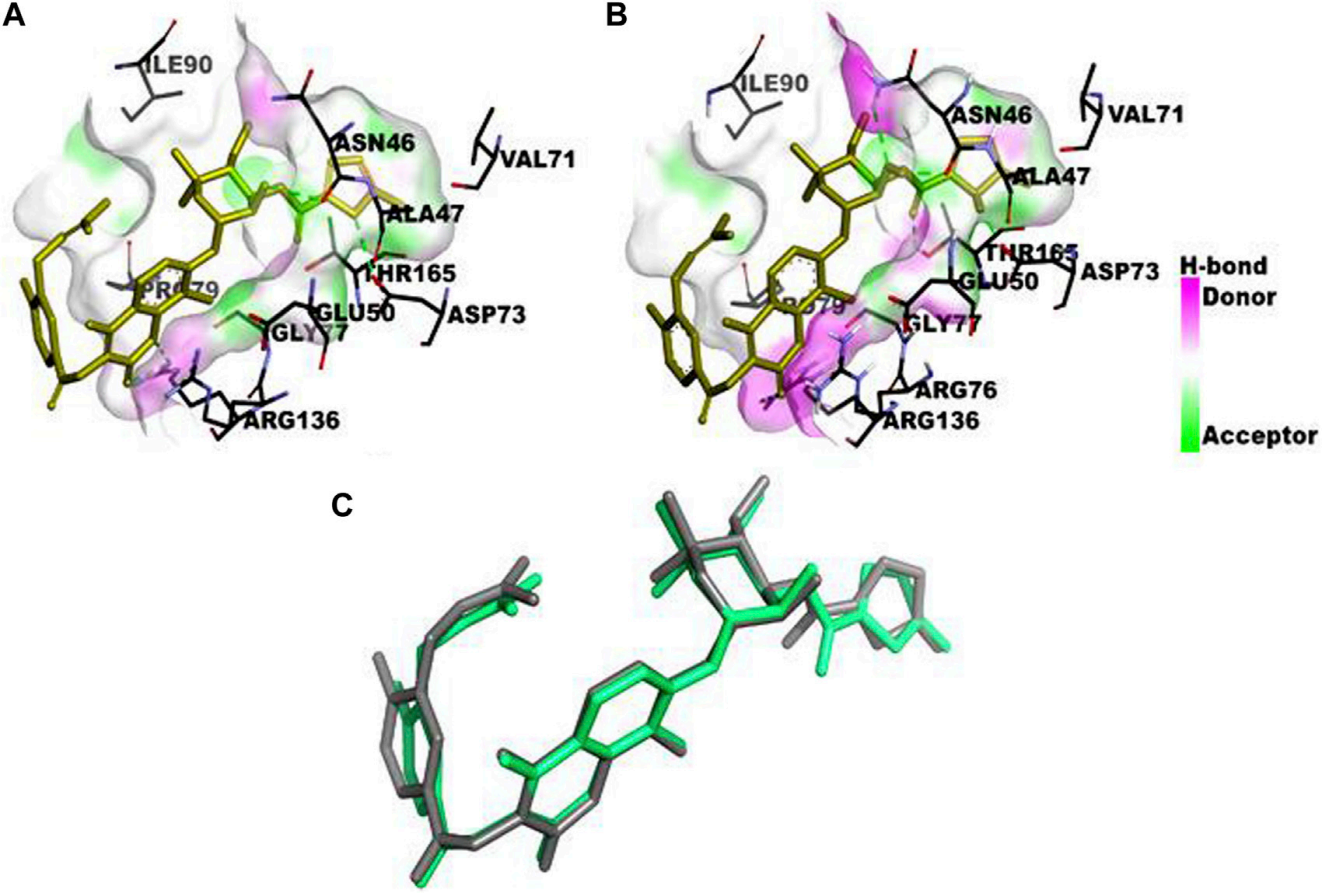
Comparison of the binding pocket of the cognate ligand clorobiocin in **(A)** the crystallized form of DNA gyrase (PDB Code: 1KZN) and **(B)** the computationally docked complex. Three of the six analyzed ligands occupy the same binding pocket **(C)**. Structural superposition of the crystallized form (green stick) and docked form (gray stick) of the ligand.

**FIGURE 6:**
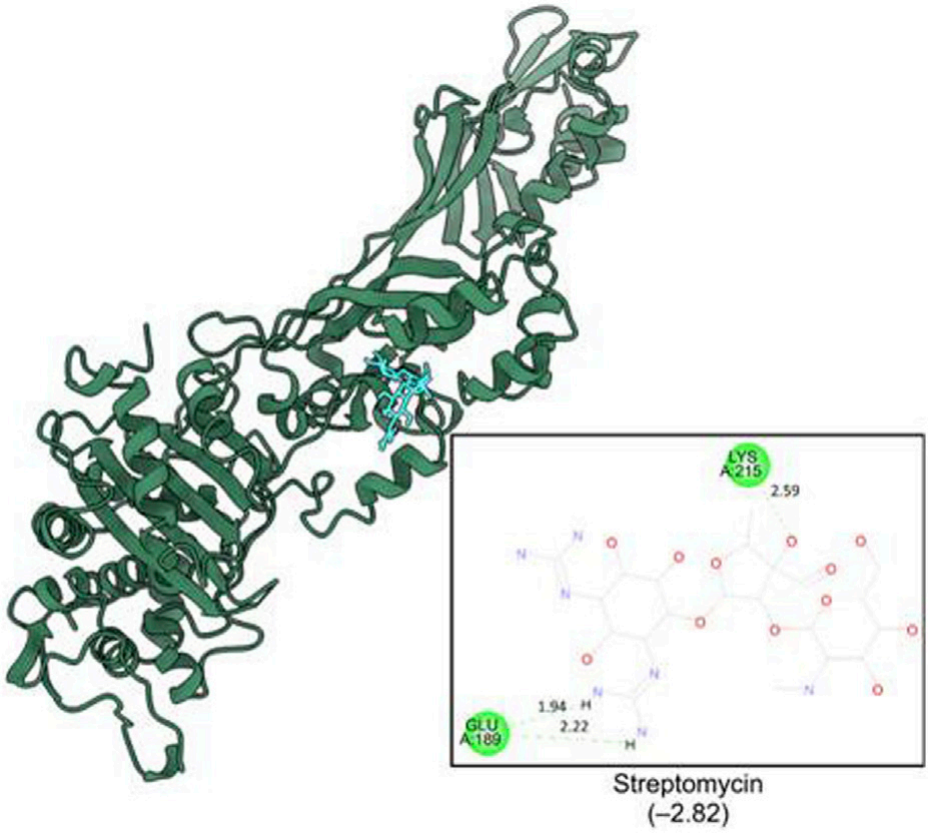
Binding pocket of streptomycin with transpeptidase (PDB id: IMWT).

**FIGURE 7:**
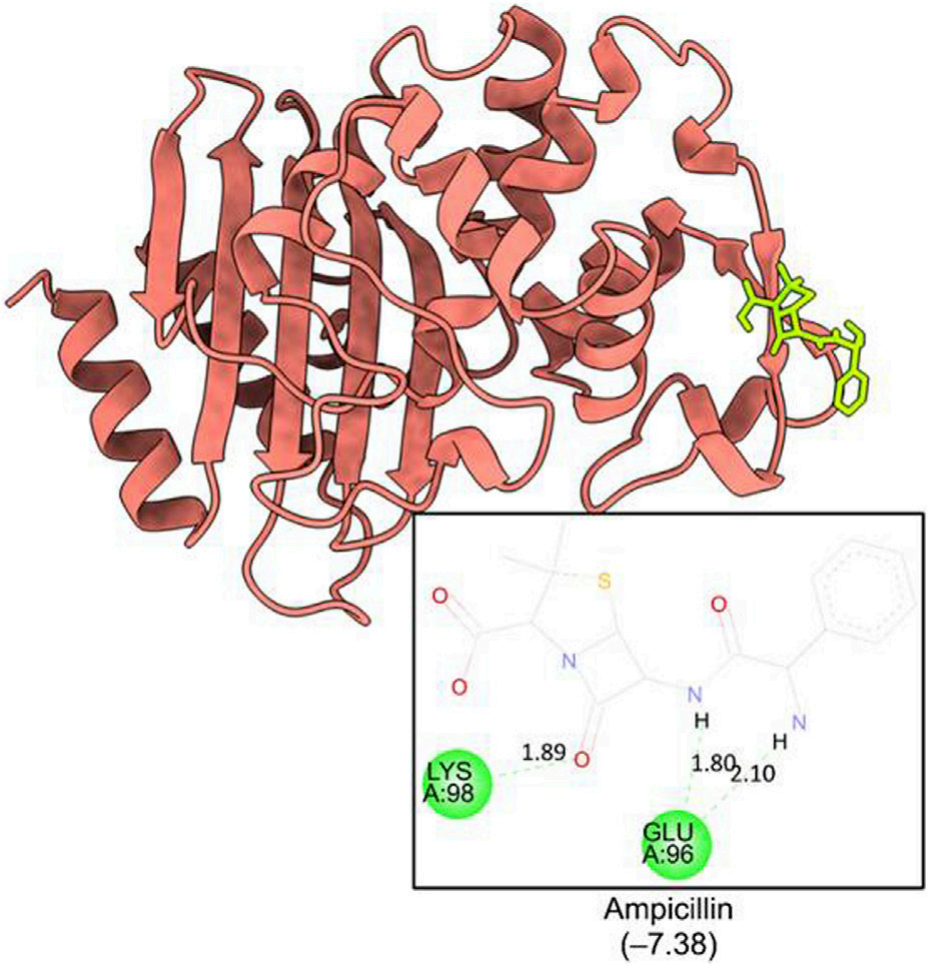
Binding pocket of ampicillin with ß-lactamase (PDB id: 6BU3).

The computational studies revealed that the cognate ligand (clorobiocin), which is an aminocoumarin antibiotic that inhibits DNA gyrase, bound to the same pocket as in the experimentally-crystallized complex (PDB Code: 1KZN), with a good docking score (−9.1 kcal/mol, Figure 5). This confirmed the robustness of the adopted docking protocol for DNA gyrase*.* This pocket is an ATP-binding site, and clorobiocin is a known competitive ATP inhibitor. Furthermore, streptomycin showed −2.82 kcal/mol of binding energy against the target enzyme, transpeptidase (Figure 6), and ampicillin showed a binding score of −7.38 kcal/mol with β-lactamase (Figure 7).

**FIGURE 8:**
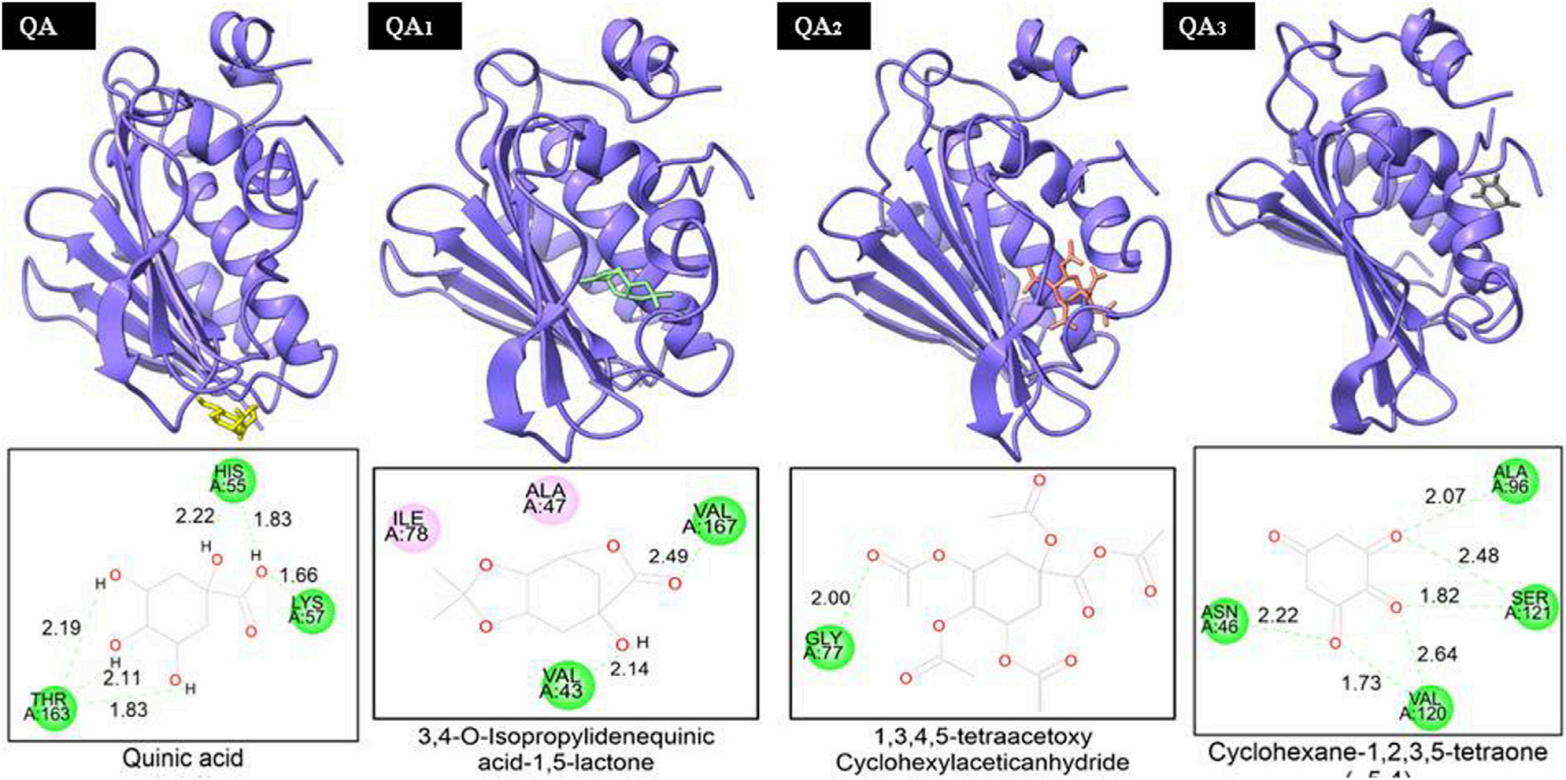
Representation of the docked structures of the complexes of DNA gyrase with the ligands quinic acid (QA), 3,4-o-isopropylidenequinic acid-1,5-lactone (QA_1_), 1,3,4,5-tetraacetoxycyclohexyla ceticanhydride (QA_2_), and cyclohexane-1,2,3,5-tetraone (QA_3_).

Two of the four ligands analyzed (compounds QA_1_ and QA_2_) docked to the same ATP-binding site as clorobiocin, with docking scores of −5.8 kcal/mol and −5.6 kcal/mol, respectively (Figure 8). This suggested that these two ligands competed for the ATP-binding site and could act as potential gyrase inhibitors. Specifically, QA_1_ interacted with VAL167 and VAL43 amino acid residues through hydrogen bonding and formed hydrophobic interactions with ILE78 and ALA47 amino acid residues that had the highest docking score (−5.8 kcal/mol) among all the ligands (Figure 8).

In contrast, the compound QA_2_ formed a single hydrogen bond with GLY77 and revealed no apparent hydrophobic contacts (Figure 6). Moreover, the remaining two ligands (compounds QA and QA_3_) bound away from the ATP-binding site, with docking scores of −4.2 kcal/mol and −5.4 kcal/mol, respectively. QA_3_ bound near the alpha-helical region of the protein and formed hydrogen bonds with ASN46, ALA96, VAL120, and SER121. QA bound near the beta sheets of the protein and formed hydrogen bonds with the HIS55, LYS75, and THR163 amino acid residues (Figure 8). None of these three ligands formed hydrophobic interactions with protein residues (Table 3).

**TABLE 3:** Molecular docking analysis of quinic acid and its derivatives against different targets.

Compounds	Docking score (kcal/mol)			Residues participating in intermolecular hydrogen bonds with ligands			Residues participating in hydrophobic contacts with ligands		
	1KZN	1MWT	6BU3	1KZN	1MWT	6BU3	1KZN	1MWT	6BU3
**QA**	−4.2	−3.65	−4.83	HIS55, LYS75, THR163	HIS251, TYR496	LYS137, GLU96, LYS98	-	LYS281	-
**QA**_**1**_	−5.8	−4.70	−6.30	VAL167, VAL43	ASN393, GLN396, GLY282	GLU96, LYS98	ILE78, ALA47	LYS281	VAL95
**QA**_**2**_	−5.6	−3.99	−5.71	GLY77	LYS218, LYS215	GLU96, LYS98, LYS137	-	-	-
**QA**_**3**_	−5.4	−4.57	−5.57	ASN46, ALA96, VAL120, SER121	SER72, ILE142, ARG65	ILE173, ARG164, THR171	-	-	-

DNA gyrase (PDB code: 1KZN), transpeptidase (PDB code: 1MWT), and β-lactamase (PDB code: 6BU3).

**FIGURE 9:**
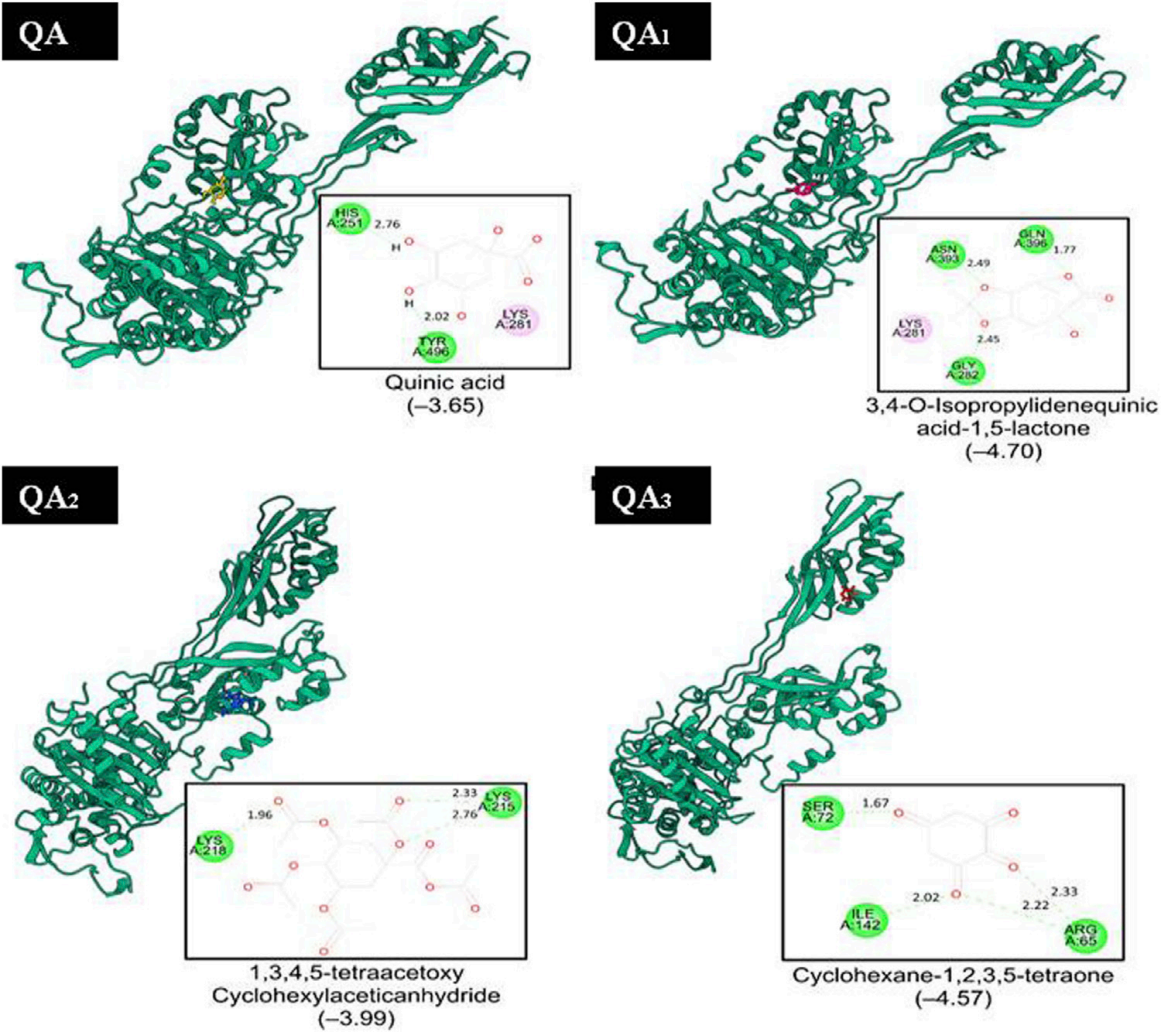
Representation of the docked structures of the complexes of transpeptidase with the ligands quinic acid (QA), 3,4-o-isopropylidenequinic acid-1,5-lactone (QA_1_), 1,3,4,5-tetraacetoxycyclohexyla ceticanhydride (QA_2_), and cyclohexane-1,2,3,5-tetraone (QA_3_).

**FIGURE 10:**
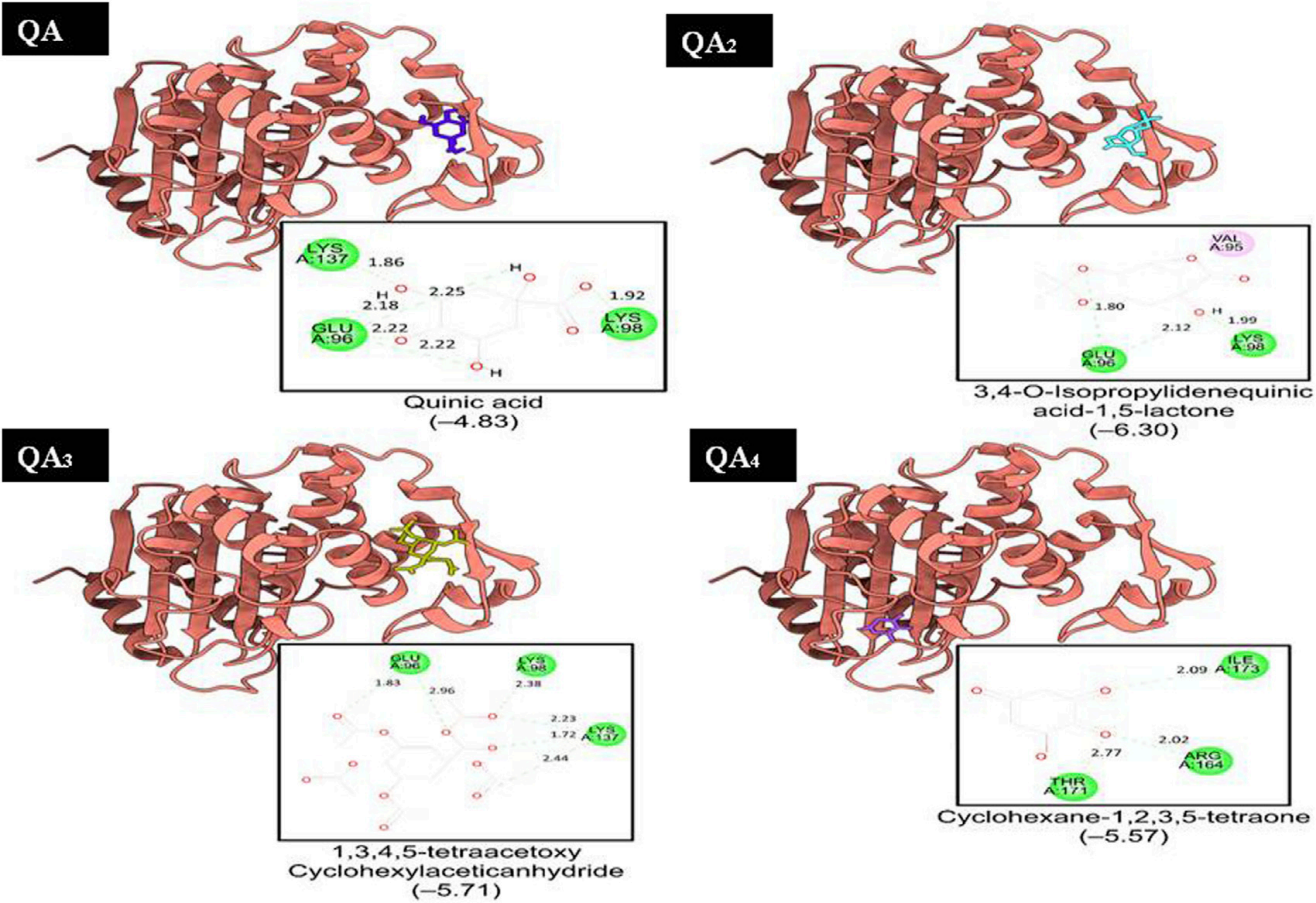
Representation of the docked structures of the complexes of ß-lactamase with the ligands quinic acid (QA), 3,4-o-isopropylidenequinic acid-1,5-lactone (QA_1_), 1,3,4,5-tetraacetoxycyclohexylacetic anhydride (QA_2_), and cyclohexane-1,2,3,5-tetraone (QA_3_).

QA_1_ also showed a binding score of −4.70 kcal/mol against transpeptidases, which was more than that of streptomycin (2.82 kcal/mol). QA_1_ formed three hydrogen bonds with ASN393, GLN396, and GLY282 amino acid residues and one hydrophobic interaction with LYS281 amino acid residues of transpeptidase (Figure 9). QA_1_ also showed a binding score of −6.30 kcal/mol against β-lactamase, which was less than that of ampicillin (−7.38 kcal/mol). QA_1_ formed two hydrogen bonds with GLU96 and LYS98 and one hydrophobic interaction with the VAL95 amino acid residues of β-lactamase (Figure 10). However, the remaining compounds showed comparatively less binding energy in the range of −3.65 to −4.57 kcal/mol against transpeptidase and −4.83 to −5.57 kcal/mol against β-lactamase. Among all the three targets tested, QA_1_ showed the maximum binding energy against β-lactamase followed by DNA gyrase and transpeptidase and, hence, justified its mode of action.

It has been reported that QA isolated from *Lonicerae Japonicae Flos* regulated core targets (rhlA, rhlR, and rhlB) in a quorum sensing system, as validated by molecular docking studies (Lu et al., 2021).

In all the docking figures, purple cartoons represent the 3D structures of the docked protein, where the ligands have been shown in different colors. Square boxes depict the residues around each ligand. Amino acid residue in the green interacted with the ligand through hydrogen bonds (hydrogen bonding distance in Å), while amino acid residues in pink were involved in hydrophobic interactions with ligands.

### 3.7 Toxicity analysis

Current toxicity studies of drug molecules using computational simulation are cost-effective, less time-consuming, and do not involve animals. The ProTox-II webserver includes both chemical and molecular target knowledge. This platform provides information about six different toxicity classes. Class 1 represents highly toxic substances (LD_50_ ≤ 5), Class 2 also includes “fatal if swallowed” and comparatively less toxic substances (5 < LD_50_ ≤ 50), Class 3 marks “toxic if swallowed” substances (50 < LD_50_ ≤ 300), Class 4 is “harmful if swallowed” substances (300 < LD_50_ ≤ 2000), Class 5 is “may be harmful if swallowed” substances (2000 < LD_50_ ≤ 5,000), and Class 6 includes substances with a non-toxic nature (LD_50_ > 5,000) (Banerjee et al., 2018).

In the present work, the compounds (I–VI) belonged to toxicity classes 3–6, as observed from ProTox-II analysis. The compounds QA, QA_1_, and QA_2_ belonged to class 6 toxicity and, hence, are safe. They may be used as future pharma drugs. The compound QA_3_ belonged to class 4, having intermediate toxicity (Table 4).

**TABLE 4:** Toxicity analysis of quinic acid and its derivatives.

Compounds	LD_50_ (mg/kg)	Toxicity class
**QA**	9,800	Class 6
**QA**_**1**_	8,000	Class 6
**QA**_**2**_	7,000	Class 6
**QA**_**3**_	2000	Class 4

### 3.8 Physiochemical properties

The term physiochemical refers to “drug-likeness” that determines the capacity of a molecule to befall as an oral drug regarding its bioavailability. It is an important step in the drug development stage after discovery and involves detailed studies on the drug formulation, stability, pharmacokinetics, metabolism, and toxicity (Ursu et al., 2011). This concept is usually employed to filter chemical libraries to exclude molecules with properties of incompatible behavior and to take forward molecules found in primary screening with an acceptable pharmacokinetics profile (Ursu et al., 2011).

The distinction between drug-like and non-drug-like properties of molecules is based on their certain physiochemical properties, which are generally evaluated by Lipinski’s Rule of Five (Borul and More, 2022). Lipinski’s Rule of Five comprises ranges of several physiochemical properties that make a molecule orally available (Ntie-Kang et al., 2019).

The physiochemical properties of all the compounds were found to be in the required range, except for a few violations, as shown in Table 5. The permeability of the drug molecule through the cell membrane is affected by various factors such as molecular weight and topological polar surface area (TPSA). In this study, the molecular weight of all the molecules fell within the required range (100–600 Da), and TPSAs of all the compounds except QA_2_ were found to be in the required range (0–140), indicating their *in vivo* permeability potential (Table 5). Another parameter is log P, which measures lipophilicity and is defined as the logarithm of the partition coefficient between the aqueous and organic phases. A value of log *p* less than 5 is favorable for a good rate of absorption (Shukla et al., 2014). In this study, log *p* values of all four compounds were in the desirable range (less than 5; Table 5). In addition, the capability of the drug molecule to cross the cell’s bilayer membrane is determined by the number of hydrogen bond donors (preferably ≤5) and acceptors (preferably ≤10). In this study, hydrogen bond donors and acceptors for all four compounds were in the acceptable range.

**TABLE 5:** Comparison of the physiochemical properties of compounds.

S. No.	Drug-likeness properties	Compounds			
		QA	QA_1_	QA_2_	QA_3_
**1**	**MW (Daltons)**	192.06	214.08	402.12	140.01
**2**	**nRig**	7	14	12	8
**3**	**f Char**	0	0	0	0
**4**	**n Het**	6	5	11	4
**5**	**Max Ring**	6	10	6	6
**6**	**n Ring**	1	3	1	1
**7**	**n Rot**	1	0	11	0
**8**	**TPSA (Å)**	118.22	64.99	148.57	74.6
**9**	**n HD**	5	1	0	2
**10**	**n HA**	6	5	11	4
**11**	**Log D (mol/L)**	−1.48	1.14	0.75	−0.48
**12**	**Log S (mol/L)**	−0.39	−1.22	−1.76	−1.11
**13**	**Log P (mol/L)**	−1.94	0.54	0.57	−0.36

Molecular weight (MW, 100–600); number of rigid bonds (nRig, 0–30); formal charge (f Char, −4–4); number of heteroatoms (n Het, 1–15); number of atoms in ring (Max Ring, 0–18); number of rings (n Ring, 0–6); number of rotatable bonds (n Rot, 0–11); topological polar surface area (TPSA, 0–140); hydrogen donors (n HD, 0–7); hydrogen acceptors (n HA, 0–12); Log D (1–3); logarithm of solubility (Log S, −4 to –0.5 log mol/L); logarithm of partition coefficient (Log P, <5).

Apart from the Lipinski parameters, the log S value determines the drug solubility, and the lowest value is preferred with the optimal range of −4 mol/L to 0.5 log mol/L. Herein, all four compounds showed the log S values in the desirable range from −1.8 mol/L to −0.3 log mol/L. Log D represents the distribution coefficient and influences the lipophilicity of ionizable compounds as it impacts the permeability property of drugs. Herein, all the compounds showed log D values between 1 and 3 as the standard limit except QA and QA_3_, which showed values of −1.48 and −0.48, respectively.

The oral bioavailability potential of drug molecules is reflected by the number of rotatable bonds with an acceptable range of ≤10 (Leeson and Springthorpe, 2007; Pollastri, 2010). In this study, compound QA showed one rotatable bond (nRot), QA_1_ and QA_3_ exhibited no rotatable bonds, and QA_2_ showed 11 rotatable bonds (Table 5), thus reflecting all the molecules have a strong potential of oral bioavailability except QA_2_ with comparatively less oral bioavailability potential.

Too many rigid bonds (nRig) (0–30) can lead to increased molecular complexity, ultimately affecting a substance’s oral bioavailability. Herein, all the compounds showed 6–14 nRig bonds. Enzymes involved in drug metabolism can interact with charged sites and further affect the normal distribution of drug molecules in the target body; therefore, the formal charge (f Char) limit is −4 to +4, and all the compounds showed zero formal charge (f Char), which is within permissible limits. More heteroatoms (nHet) indicate increased reactivity and more potential for specific types of interactions with a permissible limit of 1–15, and all the compounds showed nHet values within the range (Table 5).

**FIGURE 11:**
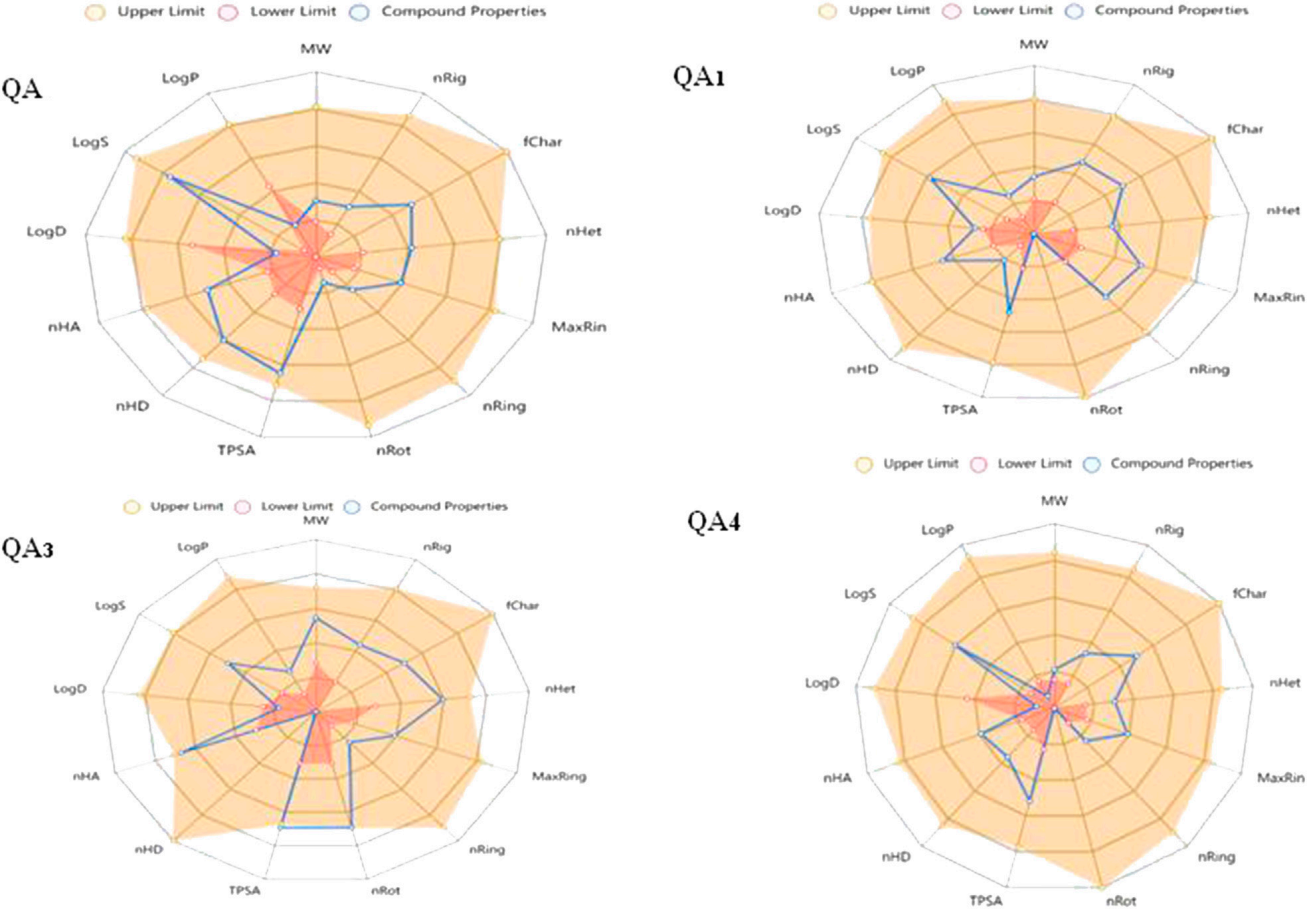
Radial plots of drug likeliness properties of compounds.

The safety limit profiles of all the physiochemical properties of compounds were studied using *in silico* approaches represented by radial plots of each compound showing the upper limit (yellow region), lower limit (red area), and compound properties (blue line). Ideally, the blue line should lie outside the red region and inside the yellow region (Figure 11).

### 3.9 ADMET analysis

All the compounds were further evaluated by *in silico* ADMET analysis to predict their pharmacokinetic profile (Van De Waterbeemd and Gifford, 2003). The natural product score (NP score) typically ranges from −5 to +5, with positive scores indicating higher natural product-likeness and negative scores indicating lower natural product-likeness (Ekins et al., 2002). In the present results, all the compounds showed values in the limited range, showing their natural product nature (Table 6). The adsorption parameters include the Caco-2 permeability property with an optimal value of > −5.15, which depicts the absorption of orally administered drugs. In the present study, all the compounds showed permeability values in the required range, except compounds QA (−6.11) and QA_3_ (−5.46); however, compound QA_1_ showed maximum value (−4.79), revealing maximum permeability. Furthermore, human intestinal absorption (0–1) was in the range of 0.0013–0.937. The distribution parameter includes the blood–brain barrier (BBB), which is the ability of a drug to penetrate the blood–brain barrier. It is a crucial factor in the distribution phase of ADMET analysis, specifically concerning the ability of a drug to penetrate the central nervous system (CNS) and reach a target site in the brain. Herein, all the compounds showed probability in the range of 0.164–0.733, and QA_1_ showed the highest (0.733) probability, suggesting higher distribution capacity.

**TABLE 6:** ADMET properties of compounds.

S. No.	Properties	Compounds			
		QA	QA_1_	QA_2_	QA_3_
**1**	**Lipinski rule**	Accepted	Accepted	Accepted	Accepted
**2**	**NP score (−5–5)**	2.24	1.54	1.61	1.25
**3**	**Caco-2 permeability (>−5.15)**	−6.11	−4.79	−4.99	−5.46
**4**	**Human intestinal**	0.89	0.008	0.937	0.159
	**Absorption (0–1)**				
**5**	**Blood–brain barrier (0–1)**	0.170	0.733	0.164	0.279
**6**	**CYP450 2C9**	0.068	0.087	0.044	0.507
	**Substrate (0–1)**				
**7**	**CYP450 2C9**	0.002	0.008	0.001	0.178
	**Inhibitor (0–1)**				
**8**	**CYP450 2D6**	0.107	0.229	0.078	0.157
	**Substrate (0–1)**				
**9**	**CYP450 2D6**	0.009	0.005	0.87	0.047
	**Inhibitor (0–1)**				
**10**	**CYP450 3A4**	0.01	0.344	0.354	0.13
	**Substrate (0–1)**				
**11**	**CYP450 3A4**	0.017	0.014	0.053	0.007
	**Inhibitor (0–1)**				
**12**	**Clearance (CL) (5–15)**	1.55	10.06	2.50	1.44
**13**	**Oral acute toxicity (0–1)**	0.011	0.077	0.017	0.373
**14**	**Skin sensitization (0–1)**	0.031	0.071	0.83	0.923
**15**	**Eye corrosion (0–1)**	0.003	0.003	0.96	0.715
**16**	**Eye irritation (0–1)**	0.282	0.04	0.58	0.99
**17**	**Respiratory toxicity (0–1)**	0.019	0.014	0.003	0.962

Cytochrome P450 enzymes (CYP enzymes) play a crucial role in drug metabolism in humans. Ideally, antibiotics should be substrates of CYP enzymes and should be metabolized by these enzymes. The antibiotic should be efficiently processed and eliminated from the body, allowing for predictable pharmacokinetics. Antibiotics that do not strongly inhibit CYP enzymes are preferred. Non-inhibitory antibiotics are less likely to interfere with the metabolism of other drugs, reducing the risk of harmful drug interactions (Anzenbacher and Anzenbacherova, 2001). All the compounds showed varied probabilities of substrate rate in different isoforms of the CYP450 enzyme; however, QA_1_ showed the highest substrate probability, suggesting its good metabolic rate as compared to other compounds. In this study, none of the compounds showed an inhibitory effect on the cytochrome P450 family of proteins, as all compounds showed a probability close to 0 except QA_3_, which showed a comparatively high probability (0.87) of being a CYP450 2D6 inhibitor, as shown in Table 6.

The excretion parameter clearance (CL) was studied; its optimum range (5–15) describes a moderate elimination of drugs from the body. In the present study, all the compounds showed values in the limited range. The toxicity parameters include oral toxicity, skin sensitization, eye corrosion, eye irritation, and respiratory toxicity, and the most effective substance, QA_1_, showed the probabilities as 0.077, 0.071, 0.003, 0.04, and 0.014, respectively. Some compounds showed a probability close to 1, indicating a likelihood of being toxic (Table 6).

## 4 Conclusion

Of QA and its derivatives, 3,4-o-isopropylidenequinic acid-1,5-lactone (QA_1_) showed maximum antibacterial potential through damage to the bacterial cell membrane along with inhibition of biofilm formation and showed synergistic interaction with streptomycin. The compound belongs to class 6 toxicity and is safe to use. The compound also has suitable physiochemical properties and pharmacokinetic profile and fulfills all the drug likeliness parameters. Hence, it can be categorized as a potential antibacterial drug candidate. However, its potential use in animal infection models needs to be determined in future *in vivo* studies.

## Data Availability

The original contributions presented in the study are included in the article/Supplementary Material; further inquiries can be directed to the corresponding author.
