# Supplementary material for: Isolation of quinic acid from dropped Citrus reticulata Blanco fruits: its derivatization, antibacterial potential, docking studies, and ADMET profiling
Source: Front Chem. 2024 Apr 18;12:1372560. doi: 10.3389/fchem.2024.1372560 (PMC11064019; doi:10.3389/fchem.2024.1372560)
Supplement: Supplementary file 1 [file DataSheet1.docx]

**Table1S: Column chromatography of ethyl acetate fraction**

| **S. No.** | **Eluent (ml)** | **Weight (g)** | **TLC based remarks** |
| --- | --- | --- | --- |
| **1.** | H_2_O: methanol (80:20) | 0.25 | Mixture |
| **2.** | H_2_O: methanol (70:30) | 0.12 | Mixture |
| **3.** | H_2_O: methanol (60:40) | 0.52 | Mixture |
| **4.** | H_2_O: methanol (50:50) | 0.68 | Mixture |
| **5.** | H_2_O: methanol (40:60) | 1.05 | Mixture |
| **6.** | H_2_O: methanol (30: 70) | 2.85 | Mixture (3 spots)  Rf = 0.31, 0.39, 0.55 |
| **7.** | H_2_O: methanol (20: 80) | 1.10 | Mixture (3 spots)  Rf = 0.33, 0.39, 0.52 |
| **8.** | H_2_O: methanol (10:90) | 0.10 | Mixture |
| **9.** | H_2_O: methanol (0:100) | 0.05 | Mixture |

**Table 2S: Column chromatography of fractions**

| **S. No.** | **Eluent (ml)** | **Weight (g)** | **TLC based remarks** |
| --- | --- | --- | --- |
| **1.** | B:A:W (1:1:3) | 0.61 | Mixture |
| **2.** | B:A:W (2:1:2) | 0.90 | Mixture |
| **3.** | B:A:W (3:1:1) | 0.48 | Single spot  Rf = 0.55 |
| **4.** | B:A:W (4:1:0) | 0.62 | Single spot  Rf = 0.57 |
| **5.** | B:A:W (5:0:0) | 0.01 | - |

**TABLE 3S: Antibacterial activity of compounds in terms of diameter of inhibition zone against *E. coli***

| **Compounds** | **Diameter of Inhibition zone (mm) at different concentrations (µg/mL)** | | | | | | | | | | | |
| --- | --- | --- | --- | --- | --- | --- | --- | --- | --- | --- | --- | --- |
|  | **100** | **150** | | **250** | | **500** | | **1000** | | | **1500** | **Mean** |
| **QA** | 0.0±0.01 | 0.0±0.01 | | 0.0±0.01 | | 10.0±0.50 | | 14.71±0.81 | | | 18.04±0.5 | 9.30^a^ |
| **QA_1_** | 0.0±0.01 | 2.50±0.50 | | 9.30±0.76 | | 17.83±0.28 | | 30.66± 1.75 | | | 33.10±0.76 | 17.12^d^ |
| **QA_2_** | 0.0±0.00 | 0.0±0.00 | | 7.16±0.76 | | 13.80±1.04 | | 19.16±0.76 | | | 24.76±0.92 | 14.50^b^ |
| **QA_3_** | 0.0±0.05 | 3.06±0.70 | | 6.00±0.50 | | 15.0±0.50 | | 23.11±1.05 | | | 27.2±1.15 | 16.01^c^ |
| **Streptomycin (positive control) (10µg/mL)** | 41.0± 0.16 | | | | | | | | | | | 41.0 |
| **Mean** | 0.0^a^ | | 1.05^b^ | | 4.60^c^ | | 12.70^d^ | | 22.40^e^ | 31.80^f^ | |  |

Data has been expressed as mean±standard deviation. Mean values followed with different superscripts in the same row of above table are significantly different (p<0.05) using Tukeys-B test

**TABLE 4S:** **Antibacterial activity of compounds in terms of diameter of inhibition zone against *Bacillus sp.***

| **Compounds** | **Diameter of Inhibition zone (mm) at different concentrations (µg/mL)** | | | | | | |
| --- | --- | --- | --- | --- | --- | --- | --- |
|  | **100** | **150** | **250** | **500** | **1000** | **1500** | **Mean** |
| **QA** | 0.0± 0.0 | 0.0± 0.0 | 0.0±0.01 | 4.43±0.41 | 9.83±0.76 | 16.66±0.611 | 5.20^a^ |
| **QA_1_** | 4.74±0.25 | 8.50±0.54 | 17.02±0.57 | 23.91±0.36 | 33.15±0.65 | 38.86±0.32 | 20.70^d^ |
| **QA_2_** | 0.0±0.0 | 4.06±0.51 | 10.80±0.77 | 18.50±0.5 | 25.53±0.45 | 29.30±0.43 | 15.81^c^ |
| **QA_3_** | 0.0±0.0 | 0.0±0.0 | 5.65±0.61 | 13.90±0.36 | 22.81±0.71 | 25.00±0.60 | 11.90^b^ |
| **Streptomycin (positive control) (10µg/mL)** | 44.5±0.04 | | | | | | 44.5 |
| **Mean** | 0.81^a^ | 2.53^b^ | 6.90^c^ | 13.01^d^ | 20.81^e^ | 30.10^f^ |  |

Data has been expressed as mean±standard deviation. Mean values followed with different superscripts in the same row of above table are significantly different (p<0.05) using Tukeys-B test

**TABLE 5S: Antibacterial activity of compounds in terms of diameter of inhibition zone against *Y. enterocolitica***

| **Compounds** | **Diameter of Inhibition zone (mm) at different concentrations (µg/mL)** | | | | | | |
| --- | --- | --- | --- | --- | --- | --- | --- |
|  | **100** | **150** | **250** | **500** | **1000** | **1500** | **Mean** |
| **QA** | 0.0±0.01 | 0.0±0.23 | 0.0±0.0 | 4.65±1.04 | 13.13±0.96 | 16.53±0.51 | 6.10^a^ |
| **QA_1_** | 0.0±0.0 | 5.76±0.25 | 10.5±0.45 | 17.41±0.40 | 29.10±0.96 | 32.13±0.65 | 15.82^d^ |
| **QA_2_** | 0.0±0.0 | 3.15±0.65 | 6.91±0.36 | 11.71±0.25 | 17.83±0.76 | 22.50±0.50 | 11.41^b^ |
| **QA_3_** | 0.0±0.0 | 4.00±0.51 | 8.00±0.05 | 14.00±0.5 | 20.53±0.53 | 24.61±0.52 | 13.51^c^ |
| **Streptomycin (positive control) (10µg/mL)** | 40.5±0.16 | | | | | | 40.5 |
| **Mean** | 0.0^a^ | 2.14^b^ | 13.01^d^ | 11.43^c^ | 20.10^e^ | 27.80^f^ |  |

Data has been expressed as mean±standard deviation. Mean values followed with different superscripts in the same row of above table are significantly different (p<0.05) using Tukeys-B test

**TABLE 6S:** **Antibacterial activity of compounds in terms of diameter of inhibition zone against *S. aureus***

| **Compounds** | **Diameter of Inhibition zone (mm) at different concentrations (µg/mL)** | | | | | | |
| --- | --- | --- | --- | --- | --- | --- | --- |
|  | **100** | **150** | **250** | **500** | **1000** | **1500** | **Mean** |
| **QA** | 0.0±0.0 | 0.0±0.0 | 0.0±0.0 | 4.06±0.6 | 10.70±0.75 | 16.00±0.40 | 5.67^a^ |
| **QA_1_** | 0.0±0.0 | 7.90±0.36 | 14.43±0.40 | 22.0±0.5 | 28.26±0.68 | 35.46±0.89 | 19.21^d^ |
| **QA_2_** | 0.0±0.0 | 5.23±0.49 | 8.33±0.49 | 15.60±0.52 | 24.81±0.8 | 28.86±0.77 | 14.81^c^ |
| **QA_3_** | 0.0±0.0 | 2.14±0.65 | 6.43±0.40 | 13.53±0.55 | 23.12±0.36 | 24.43±0.55 | 12.76^b^ |
| **Streptomycin (positive control) (10µg/mL)** | 42.0±0.15 | | | | | | 42.0 |
| **Mean** | 0.0^a^ | 2.55^b^ | 5.21^c^ | 11.75^d^ | 20.11^e^ | 28.95^f^ |  |

Data has been expressed as mean±standard deviation. Mean values followed with different superscripts in the same row of above table are significantly different (p<0.05) using Tukeys-B test


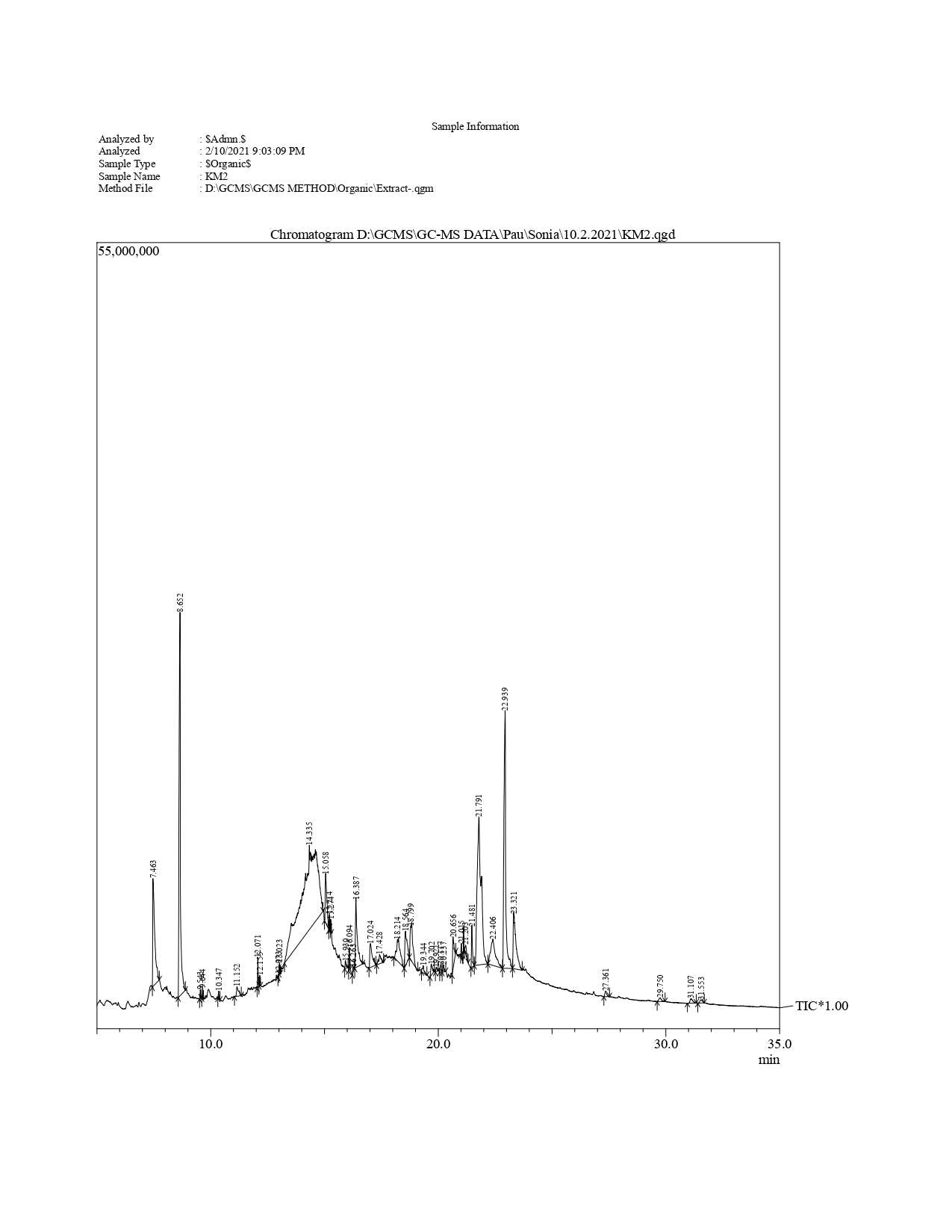


**FIGURE 1S: GC-MS of ethyl acetate fraction**


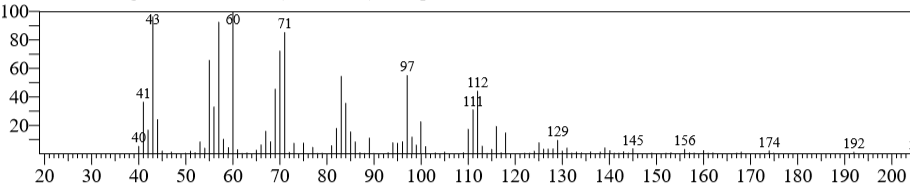


**FIGURE 2S: MS chromatogram of QA**

**
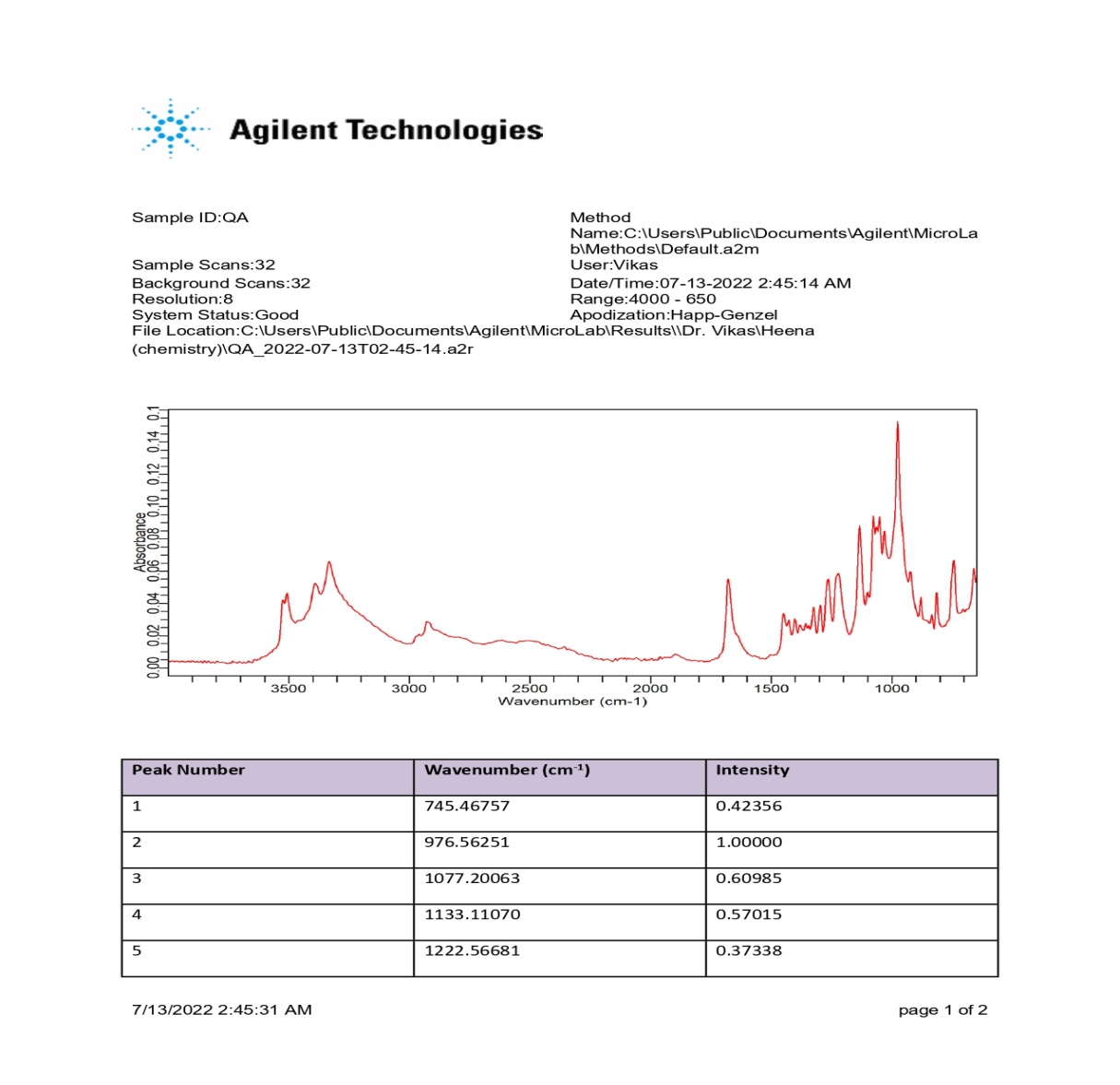
**

**FIGURE 3S: FT-IR chromatogram of QA**

**
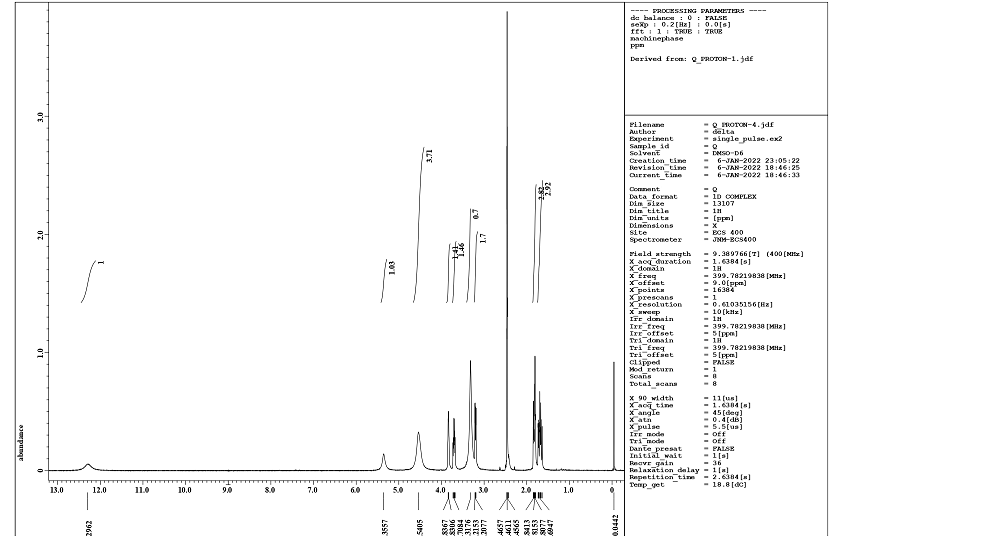
**

**FIGURE 4S: ^1^H NMR spectra of QA**

**
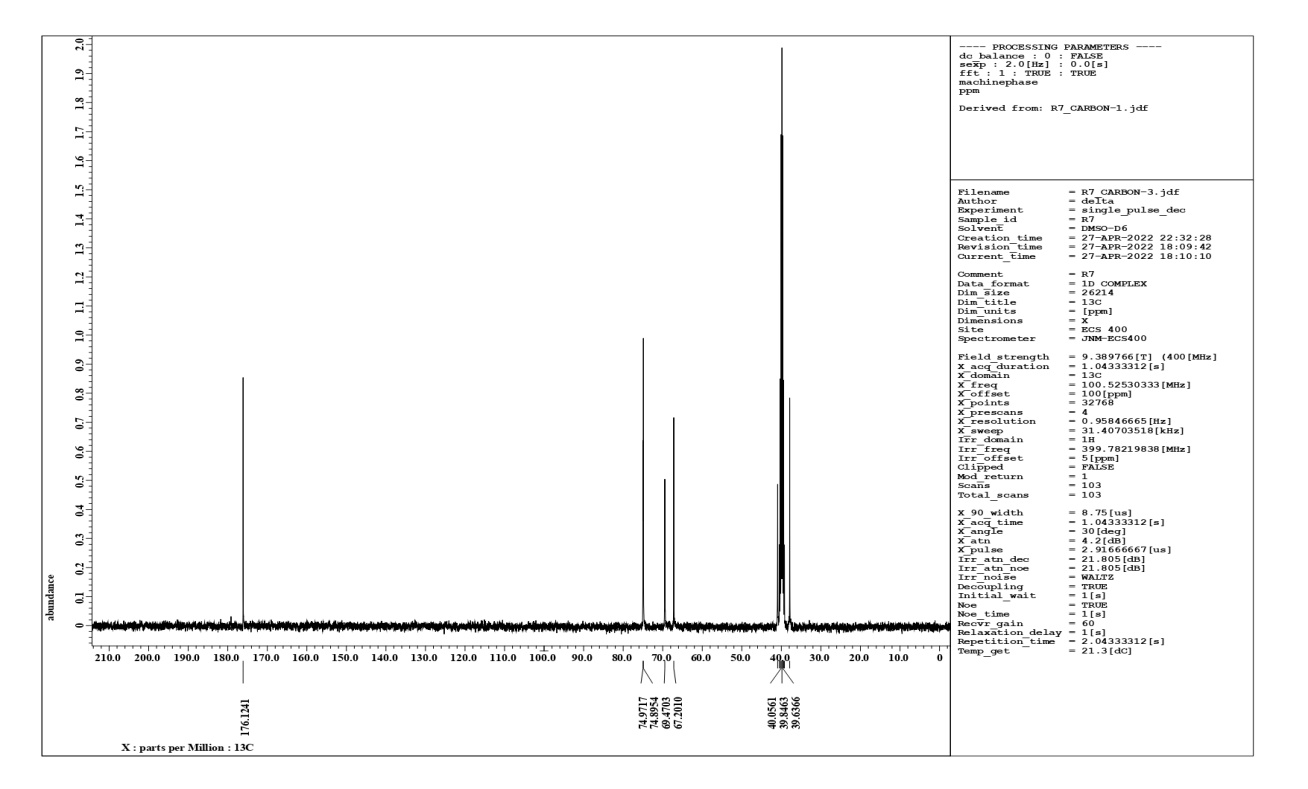
**

**FIGURE 5S: ^13^C NMR** **spectra of QA**

**
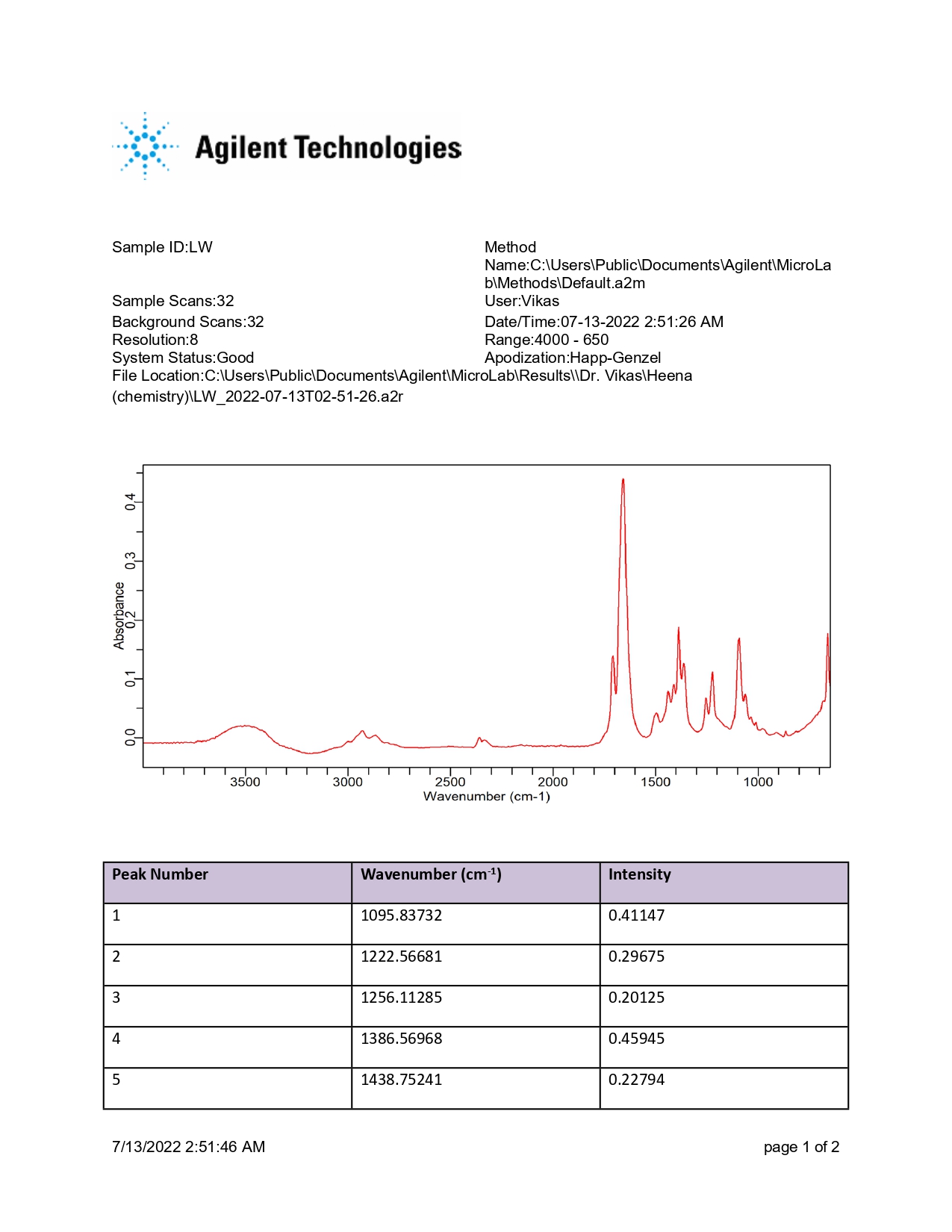
**

**FIGURE: 6S: FT-IR spectra of QA_1_**


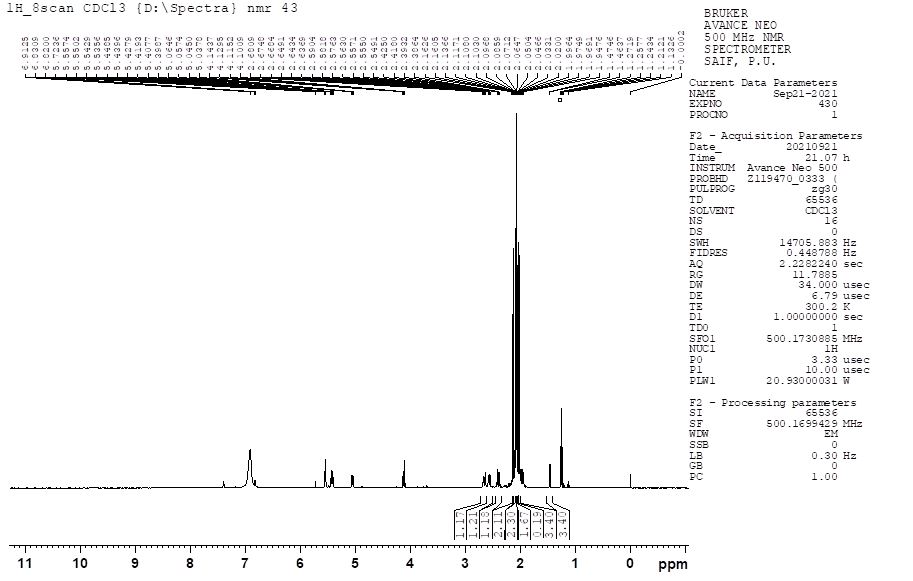


**FIGURE 7S :** **^1^H NMR spectra of QA_1_**


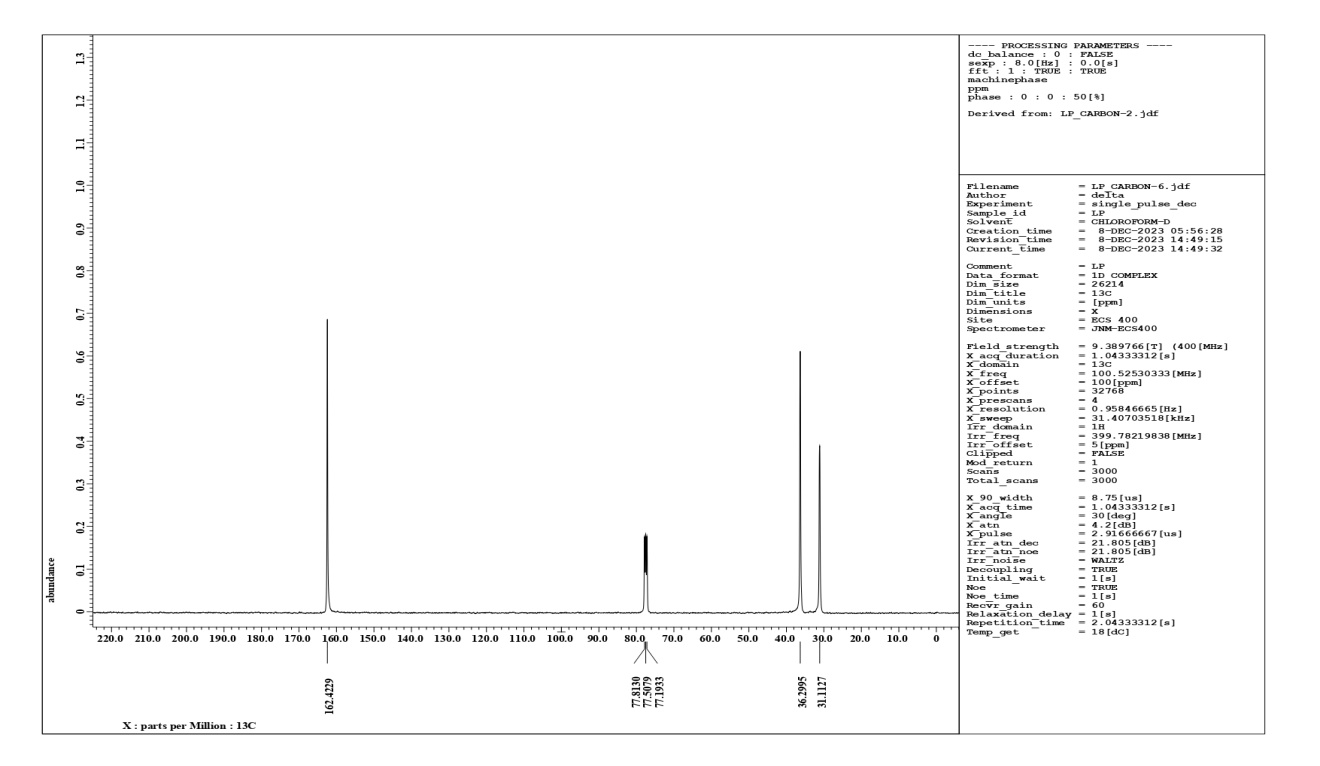


**FIGURE 8S: ^13^C NMR spectra of QA_1_**


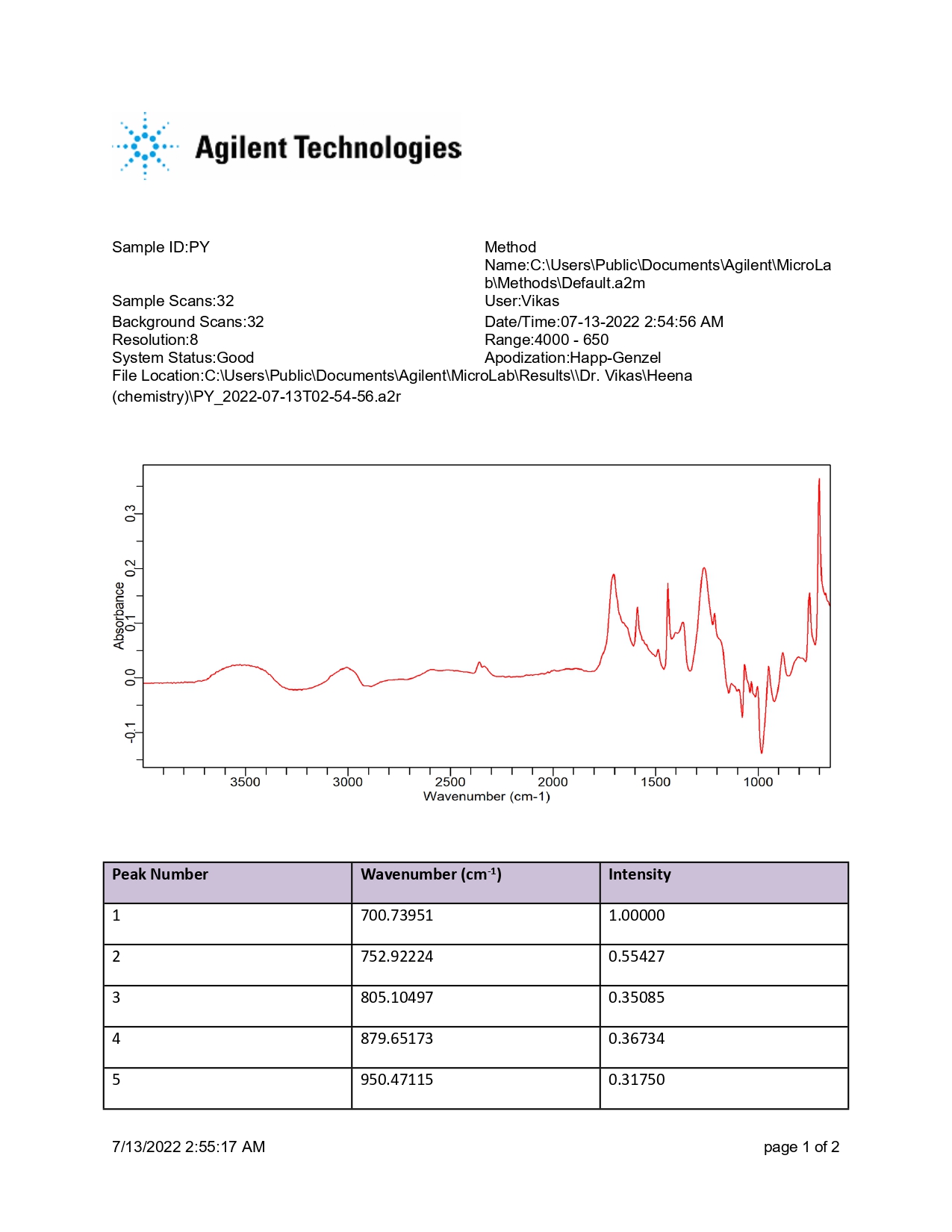


**FIGURE 9S: FT-IR spectra of QA_2_**


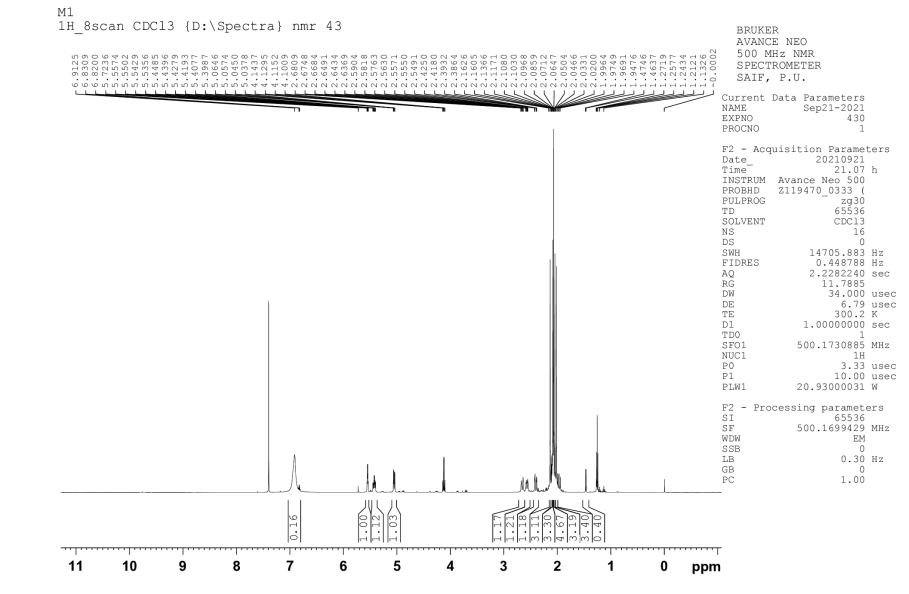


**FIGURE 10S:** **^1^H NMR spectra of QA_2_**


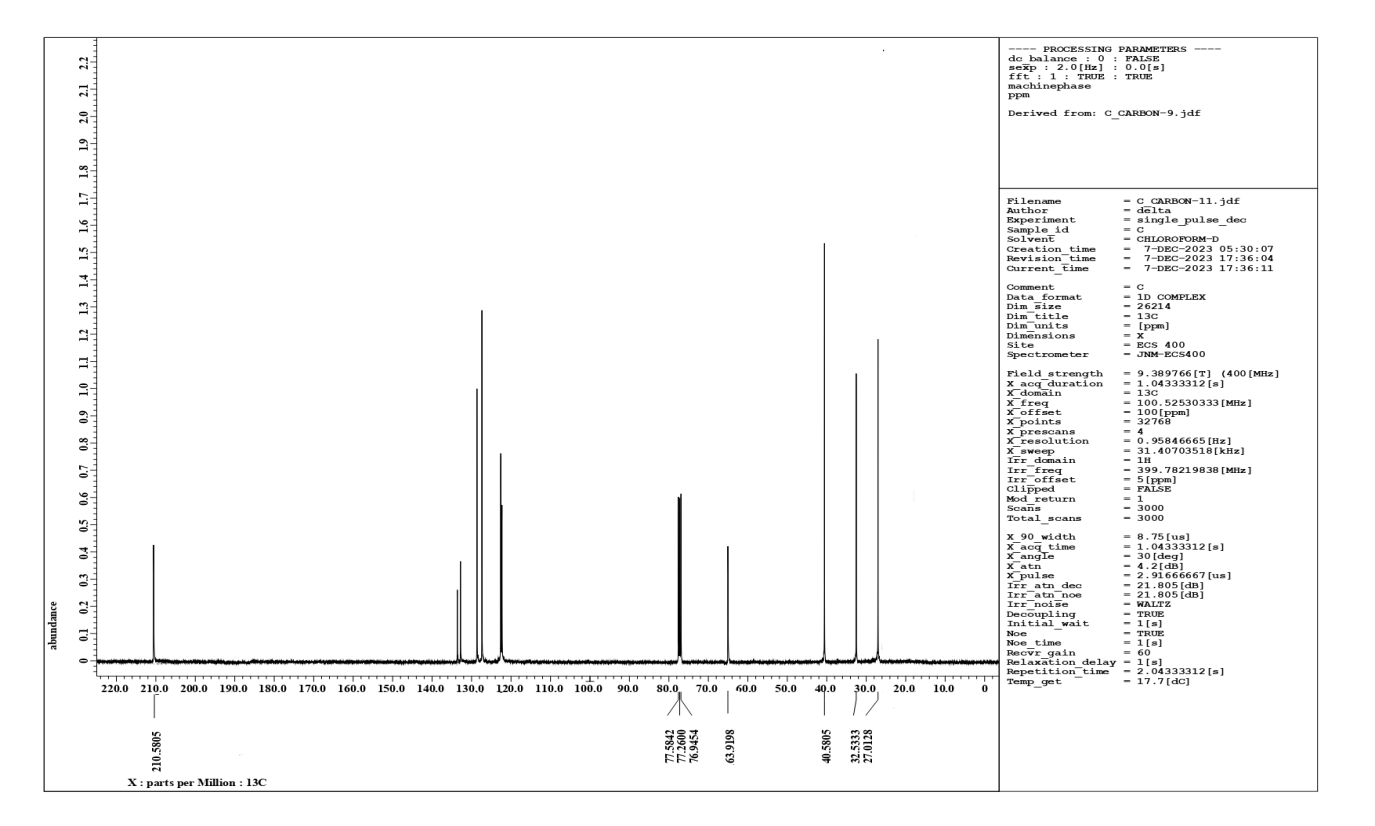


**FIGURE 11S:** **^13^C NMR spectra of QA_2_**


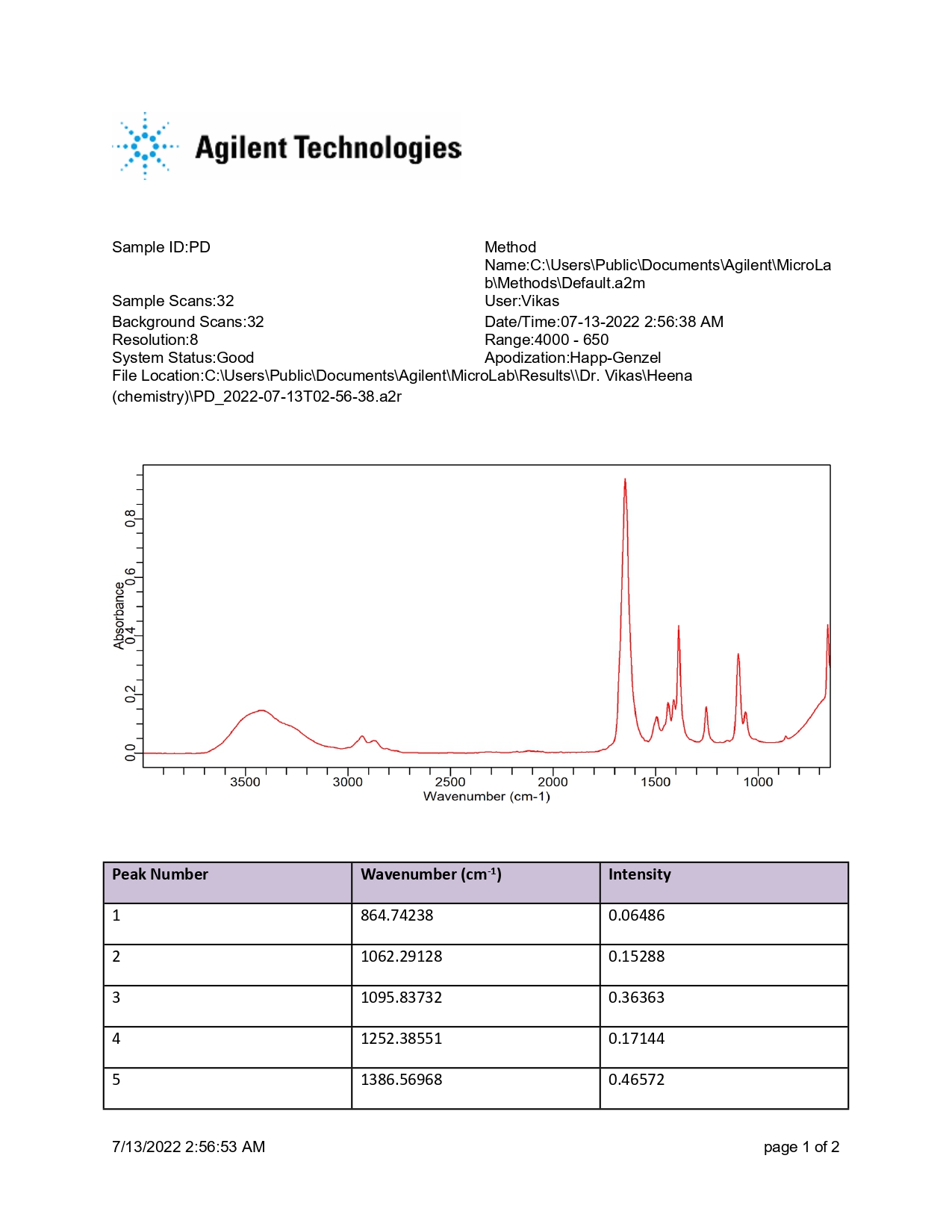


**FIGURE 12S: FT-IR spectra of QA_3_**


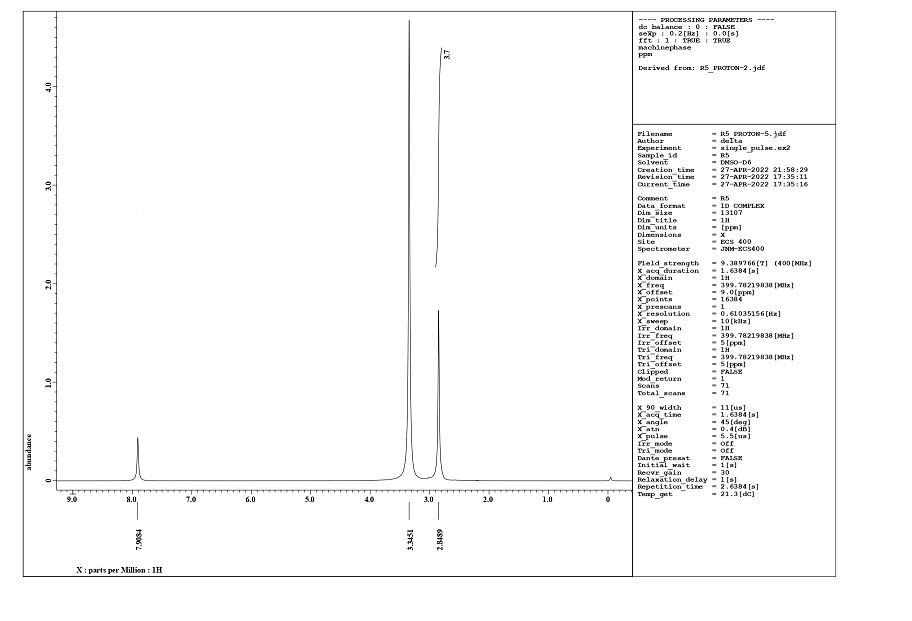


**FIGURE 13S:** **^1^H NMR spectra of QA_3_**


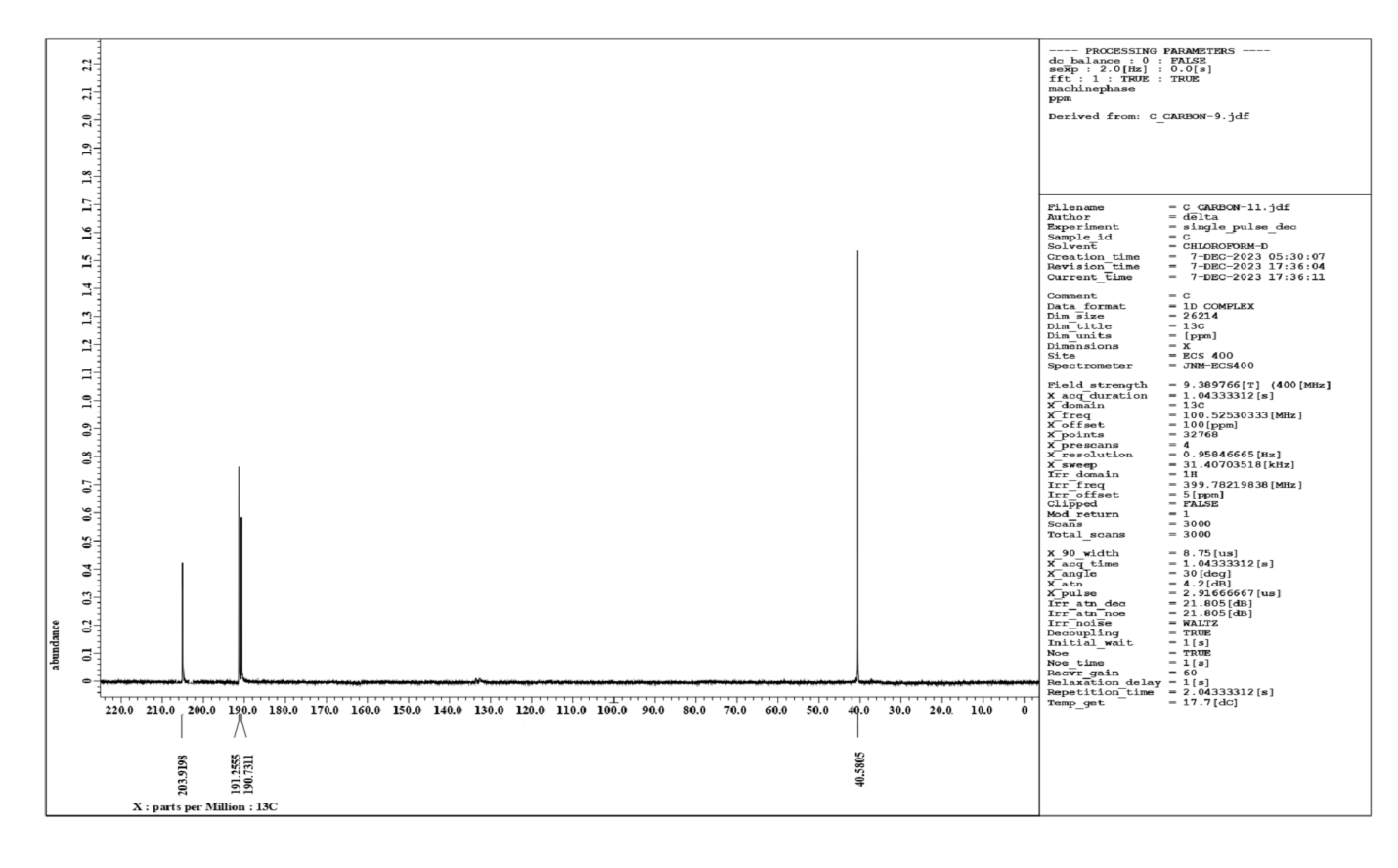


**FIGURE 14S: ^13^C NMR spectra of QA_3_**
